# Putting the Social in Emotions: The Effect of Audience Presence on Pride and Embarrassment Across Ontogeny

**DOI:** 10.1111/desc.70024

**Published:** 2025-05-19

**Authors:** Christopher Riddell, Milica Nikolić, Mariska E. Kret

**Affiliations:** ^1^ Institute of Psychology, Cognitive Psychology Unit Leiden University Leiden The Netherlands; ^2^ Leiden Institute for Brain and Cognition (LIBC) Leiden The Netherlands; ^3^ Research Institute of Child Development and Education University of Amsterdam Amsterdam The Netherlands

**Keywords:** audience effects, embarrassment, impression management, pride, socioemotional development

## Abstract

We care about others’ opinions of us and regulate our emotions to make positive impressions. This form of impression management may change during ontogeny as children become increasingly sensitive to others. To examine whether self‐conscious emotions are influenced by audience presence across the lifespan, we induced embarrassment and pride in *n* = 71 3.5–5‐year‐old children, *n* = 71 8–10‐year‐old children, and *n* = 73 adults, either in the presence of an audience or alone. We measured nonverbal expressions of emotion, physiological arousal, and self‐reported emotional experiences. All participants reported more embarrassment and blushed more while watching their singing performance in the presence of others. However, participants’ pride was not contingent on audience presence and differed across age, with adults showing the most nonverbal expressions of pride. These results elucidate how social environments shape how we feel and express emotions across development.

## 1 Introduction

The feeling of being watched by others bears significant influence on the way we behave (Kampis and Southgate 2020), likely because we care about others’ opinions of us (Baumeister 1982; Leary 2019). We bicycle faster (Triplett 1898), donate more money to causes (Satow 1975), make riskier investment decisions in economic games (Goulart et al. 2015) and cheat less (Engelmann et al. 2012) when we are observed compared to when we are alone, even in early childhood (Banerjee 2002; Haun et al. 2014; Haun and Tomasello 2011). These so‐called “audience effects”—that is, any modification in behavior attributable to the belief that another person may be watching—have captured the interests of scientists for over a century (Hamilton and Lind 2016; Triplett 1898). More recently, attention has turned to the ways in which the experience and expression of certain emotions may be impacted by the (real or imagined) presence of onlookers in the environment (Fridlund 1991; Fridlund et al. 1992; Shearn et al. 1992; van Osch et al. 2019; Webster et al. 2003). For example, adults express more joy after bowling a strike (Kraut and Johnston 1979), make more intense facial expressions after smelling unpleasant odors (Gilbert et al. 1987), and laugh more during the viewing of pleasant videotapes (Fridlund 1991) in the company of others compared to when they are alone.

**Summary**

- Children (ages 3.5–5, 8–10) and adults watched recordings of themselves singing or successfully solving a puzzle to elicit embarrassment and pride, with or without an audience.
- While watching their singing performance, participants reported more embarrassment and blushed more when an audience was present compared to when it was absent.
- Pride was unaffected by audience presence, but adults showed more nonverbal expressions of pride, and children showed greater physiological arousal while watching themselves solve the puzzle.
- These findings highlight the influence of the immediate social environment and age on how we feel and express self‐conscious emotions. 

Understanding audience effects on emotions is important because emotions are not solitary phenomena, but are rather inextricably linked to the social world (Fischer et al. 2016; Frijda and Mesquita 1994; Mesquita and Parkinson 2024; van Kleef et al. 2016) and highly influenced by situational contexts (Harris 1983; Schachter and Singer 1962). Nonverbal emotional displays communicate internal states to others (Fridlund 1991) and therefore should inevitably increase when potential communication partners are around compared to when they are absent. For example, one study found that adults’ smiling increased in the presence of an audience compared to when they were alone, although they did not report feeling their emotions more intensely (Fridlund 1991). Additionally, when others are present, people tend to engage in impression management strategies to influence others’ perceptions of their competencies, even in childhood (Asaba and Gweon 2022; Banerjee 2002; Bond 1982; Botto and Rochat 2019). For example, in relation to emotions, culturally‐specific display rules operate and guide how, and in which social contexts, certain emotions are expressed (Malatesta and Haviland 1982). For example, scrunching one's nose and down‐turning one's lips (i.e., displaying the prototypical facial expression of disgust) after tasting a meal prepared by a friend will likely result in a negative, and therefore undesirable, evaluation from one's surroundings in certain cultural contexts. This makes such a display less likely to occur in the presence of other people.

Audience effects, therefore, may reflect a more general sensitivity to the thoughts, beliefs, and judgments of people around us (Hamilton and Lind 2016). Increasing sensitivity to other people likely coincides with developing socio‐cognitive abilities across childhood, including Theory of Mind (Hamilton and Lind 2016) and understanding of social standards, rules, and goals (Leary et al. 1992). As such, exploring how emotional expressions are modulated by the presence of others across ontogeny provides an interesting avenue to pry into children's developing impression management abilities and self‐presentation more generally.

Children's reputational concerns are argued to stem from the development of two key components in early childhood—the realization that others can positively or negatively evaluate their behavior, and the default preference to want these evaluations to be positive rather than negative (Botto and Rochat 2019). Because evaluative concerns become prominent only from around the ages of four to five and increase across childhood (Nikolić et al. 2022), it is theorized that children in early childhood are less likely than older children and adults to adjust their behavior in the company of others compared to when they are alone (Botto and Rochat 2019). With increasing evaluative concerns at the end of childhood and at the emergence of adolescence (Nikolić et al. 2019, 2019; Westenberg et al. 2004), children in late childhood; however, would be expected to become more adult‐like and show sensitivities to audiences, modifying their behavior in the presence of others to manage their reputation, as adults do. This is supported by one study which found that 8‐year‐old children, but not 6‐ and 7‐year‐old children, displayed more intense facial expressions of joy and disappointment in the presence of others compared to when they were alone (Holodynski 2004). Further work that compares the effect of audience presence on the expressions and experience of a variety of emotions in children of different ages, including children in early childhood (as well as with adults), is thus needed.

### 1.1 Self‐Conscious Emotions and Audience Effects

Interestingly, although there is evidence that the expressions of basic emotions, such as happiness, sadness, fear, disgust, anger and surprise are modulated by audience presence in both adults and children (Ekman 1970, 1992; Holodynski 2004; Tracy and Weidman 2021), there has been a distinct lack of research on audience effects on complex social emotions. One class of social emotions, referred to as self‐conscious emotions, is those that are highly related to our sense of self and our awareness of others’ reactions to us (Lewis et al. 1989; Lewis 2008). This group of emotions includes pride, embarrassment, shame, guilt, and envy (Tracy and Weidman 2021). In the current study, we focus on two self‐conscious emotions, pride—as an example of a positive self‐conscious emotion—and embarrassment—as an example of a negative self‐conscious emotion. Pride is an emotion that occurs when an individual attributes success in an area that they value to their own efforts (Tracy and Robins 2004). Embarrassment, conversely, is an emotion that occurs when one violates social conventions or disrupts social relationships, both of which result in the feeling of social exposure (Lewis and Ramsay 2002). Unlike shame, another negative self‐conscious emotion that occurs as a result of more serious moral or social transgressions, embarrassment typically occurs due to minor violations, such as mishaps and social faux pas (Keltner and Buswell 1996). Self‐conscious emotions may be particularly susceptible to audience effects for two reasons. First, self‐conscious emotions are known to be elicited when the attention of others is put on the self (Leary 2004; Lewis et al. 1989; Lewis 2007), and, therefore, with increased social attention from the presence of an audience, these emotions may be experienced and expressed more intensely. Second, self‐conscious emotions are known to have the communicative function of navigating one's own reputation and relationships with other people (Sznycer 2019), and, as such, the need to communicate these emotions may be greater in the presence of others (Dijk et al. 2009; Keltner and Buswell 1997).

Self‐conscious emotions have typically been investigated in the context of social situations, and are assumed to start developing in toddlerhood and throughout childhood (Lewis et al. 1989; Stipek 1995; Tracy and Weidman 2021). At around three years old, once children have the capacity for self‐reflection and understand the rules and standards of their culture, children are argued to be able to experience embarrassment and pride (Lewis and Wolan Sullivan 2005). As these concerns become more prominent over ontogeny, it is likely that the expressions of these emotions similarly become stronger with age (Nikolić et al. 2022). As such, it is relevant to investigate not only their developmental patterns across age, but also the impact of audience presence on their expression and experience. The impact of audience on self‐conscious emotions could vary across age and may suggest differential (or different intensities) of presentation‐ and impression‐management strategies in different periods of life.

In one of the few investigations of audience effects on self‐conscious emotions, Holodynski (2006) found that 3–7‐year‐old children did not express pride and shame in solitary situations, but only in social situations, suggesting that these emotions need a social component to be expressed. Another study found that 4–10‐year‐old children displayed more negative body postures when they unintentionally disadvantaged a peer, but only in a social context, perhaps reflecting an increase in the experience of emotions such as shame or guilt (Gerdemann, McAuliffe, et al. 2022). However, given the large age ranges of the children in these works and their predominant focus on behavioral measures of emotion, new work is needed to understand whether audience presence may influence self‐conscious emotions in the same or different way in early childhood, late childhood and adulthood, considering different components of emotions. For example, not only nonverbal behaviors may be impacted by audience presence—but also physiological aspects and self‐reported experiences.

### 1.2 Audience Effects on Physiological Responding

Emotions inevitably involve changes to physiological activity, brought upon by the activity of the autonomic nervous system (Bradley and Lang 2007; Friedman and Thayer 2024). These changes likely reflect an autonomic arousal response (de Vente et al. 2014; Kreibig 2010; Nikolić et al. 2016), which involves the activation of the sympathetic and deactivation of parasympathetic nervous systems (or both) (de Vente et al. 2014; Porges 2009). The sympathetic nervous system triggers the “fight or flight” response in the body, whereas parasympathetic stimulates digestion and other homeostatic processes (de Vente et al. 2014). Increases in skin conductance level are known to reflect sympathetic activation (Dawson et al. 2007; de Vente et al. 2014), whereas decreases in high‐frequency heart rate variability are known to reflect deactivation of the parasympathetic system (Penttilä et al. 2001). Feelings of embarrassment are associated with an increase in skin conductance level and a decrease in heart rate variability (Gerlach et al. 2001; Harris 2001; Kreibig 2010). Although studies of pride are more limited, previous works have linked pride to increases in skin conductance level, while the findings on heart rate variability are more mixed (Behnke et al. 2022). Work examining the arousal response during embarrassment‐eliciting situations in childhood suggests that this pattern of responding occurs even in early childhood (Nikolić et al. 2016).

In addition to this general emotional arousal, blushing (i.e., reddening of the face and neck) is a physiological response that arises in response to social exposure (Crozier 2004; Drummond 2013; Leary 2019), and has also been demonstrated to occur during experiences of embarrassment and pride. Blushing is assumed to be a hallmark physiological response specific to self‐conscious emotions (Crozier and Jong 2012). For example, blushing occurs in response to embarrassing situations, such as performing or watching oneself perform a song, in early childhood (Nikolić et al. 2016), late childhood (Nikolić et al. 2019, 2019), and adulthood (Gerlach et al. 2001; Mulkens et al. 1997). Additionally, some studies have also reported blushing in situations that may evoke pride, such as receiving (exaggerated) compliments (Brummelman et al. 2018; Leary et al. 1992; Leary and Meadows 1991; Nikolić et al. 2018). Compared to the other peripheral measures, the physiological underpinnings of blushing are less clear (de Vente et al. 2014). However, Drummond et al. (2020) suggest that blushing reflects an increase in blood flow through superficial vessels in the face (and sometimes neck and chest) that is driven by an increased sympathetic nervous system activity. This range of physiological measures gives us valuable insights into participants’ patterns of emotional responding. These responses may be particularly interesting to investigate in the context of audience effects as they are under less volitional control compared to nonverbal emotional expressions, yet still visible (either directly or indirectly) to observers (Kret 2015).

### 1.3 Present Study

We aimed to establish whether the self‐reported experience, nonverbal expression and physiological response associated with the self‐conscious emotions pride and embarrassment are modulated by the presence of an audience across three age groups: 3.5–5‐year‐old children (representing early childhood), 8–10‐year‐old children (representing later childhood) and adults. These age ranges broadly reflect the periods of ontogeny in which self‐conscious emotions and audience sensitivity are expected to emerge (younger children) and become more adult‐like (older children) (Lewis and Wolan Sullivan 2005; Somerville et al. 2013). Of all the self‐conscious emotions, we chose embarrassment and pride because we aimed to investigate both positive and negative emotions. While pride is the only positive self‐conscious emotion, embarrassment was chosen because there is a well‐validated task known to evoke embarrassment across different ages, allowing for a direct comparison between age groups (e.g., Gerlach et al. 2001; Nikolić et al. 2019, 2019). To this end, participants watched, in a counterbalanced order, recordings of themselves previously (1) solving an age‐appropriate puzzle task, which we claimed was very difficult and receiving bogus positive feedback (regardless of their performance) and (2) singing a song of their choice in front of a camera for 1 min.

A video of participants receiving positive feedback during the puzzle task was used to elicit pride, and a video of the singing performance was used to elicit embarrassment. Receiving positive feedback is known to induce pride in both children (Stipek et al. 1992) and adults (Fourie et al. 2011; Williams and DeSteno 2008). Similarly, watching back oneself performing a song is known to elicit embarrassment in both children (Nikolić et al. 2016) and adults (Gerlach et al. 2001; Mulkens et al. 1997). Half of the participants viewed these videos completely alone, and the other half viewed these in the presence of two audience members. We recorded participants’ nonverbal expressions of pride and embarrassment, as well as their physiological response (cheek temperature, skin conductance level, heart rate variability) in the viewing phase to examine if these were modulated by the presence of the audience. Additionally, we asked older children and adults to indicate self‐reported embarrassment and pride.

We expected that both the viewing of the singing performance and the puzzle task would result in a general physiological arousal response (i.e., an increase in skin conductance level, a decrease in heart rate variability) and self‐conscious physiological arousal (i.e., an increase in cheek temperature reflecting physiological blushing) (de Vente et al. 2014; Nikolić et al. 2016). However, we hypothesized that adults would demonstrate more embarrassment during the rewatching of the singing performance (captured through both nonverbal expressions of embarrassment and physiological arousal), and more pride during the watching back of the puzzle task (captured through nonverbal expressions of pride and physiological arousal) than both older and younger children, but especially so in the presence of an audience. We expected the presence of the audience to be less important for children's emotional responding, and especially for that of young children. As such, we expected fewer nonverbal expressions of embarrassment and pride, as well as less physiological arousal in the presence of the audience in younger children compared to older children and adults (age group * audience condition interaction). This is based on the notion that the intensity of self‐conscious emotions is known to increase across childhood (Lewis 2007), with a peak in the late‐adolescent years and young adulthood (Somerville et al. 2013), and that sensitivity to audience evaluation increases from early to late childhood (Botto and Rochat 2019).

## 2 Method

### 2.1 Participants

Two‐hundred‐sixteen participants were invited to participate in the experiment: *n =* 71 younger children (*M_age_* = 4.06, *SD_age_* = 0.51, range: 3.42–5.25‐years‐old), *n* = 71 older children (*M_age_* = 8.83, *SD_age_* = 0.62, range: 7.92–10‐years‐old) and *n* = 73 adults (*M_age_* = 23.39, *SD_age_* = 4.89, range: 18–40‐years‐old). This sample size was calculated a‐priori through simulation using the Superpower package in R (Caldwell et al. 2022). We based our sample size calculation on ensuring sufficient power for a 3 (age group) * 2 (audience condition) * 2 (emotion induction task) mixed‐design ANOVA, although we deviated slightly from this analysis strategy as it was deemed unsuitable to test our hypotheses and used analysis which required smaller sample size, thus ensuring, we had at least 80% power (see Section 2.5). A sample size of *n* = 70 per age group was needed to have 80% power to detect a three‐way (age * audience condition * emotion induction task) interaction on nonverbal emotional expression duration with small‐to‐medium effect sizes (η_p_^2^ = 0.06) at the standard alpha 0.05 error probability.

All participants were fluent in Dutch or English, reported normal (or corrected‐to‐normal) vision, no current treatment for any psychological disorders, and no current use of any psychoactive medications. Children were recruited through various channels, including online advertising via social media, as well as through local schools and nurseries. Adult participants were recruited both through the Leiden University compulsory research participation program (for first‐year psychology students) and online advertising. Adult participants received research credits or were paid according to the Leiden University standard hourly rate (€7.50) for their participation. Children received a small gift bag with toys and a “Young Scientists” certificate for their participation.

**Missing data**. *n* = 5 younger children and *n* = 1 older child were excluded from the embarrassment nonverbal behavior analyses due to refusal or inability to participate in the singing task. An additional *n* = 1 adult was excluded because of a technical issue, leaving a total of *N =* 209 participants included in the final embarrassment nonverbal expression models. Additionally, a number of younger children were uncooperative and refused physiological measurement. *n* = 12 younger children and *n* = 1 older child refused the cheek temperature sensor, resulting in a total of *n* = 203 participants remaining in the cheek temperature analyses. *n* = 6 younger children refused the skin conductance level (SCL) electrodes, resulting in a total of *n* = 210 participants remaining in the SCL analyses. *n* = 8 younger children refused the electrocardiogram (ECG) electrodes, resulting in a total of *n* = 208 participants remaining in the ECG analyses.

### 2.2 Procedure

After participants arrived at the lab, we invited them to take part in a singing performance and a puzzle task, which we recorded to use as stimuli for our experiment. Participants were told that a camera and a webcam would be used to record their faces and bodies for the analysis of their nonverbal emotional expressions. For the experiment, participants rewatched, in counterbalanced order, the videos of themselves (1) solving a puzzle and receiving compliments, used in previous works to induce pride (Fourie et al. 2011; Stipek et al. 1992) and (2) performing a song, used in previous works to induce embarrassment (Gerlach et al. 2001; Nikolić et al. 2016). Participants’ behavioral and physiological responses were recorded during the viewing task. We opted to measure responses only during the viewing phase to ensure that we could manipulate audience presence in the same way for all age groups: younger children could not be left alone in a room to complete a puzzle and perform a song, which would have made examining the impact of audience presence on these emotions impossible. We randomly assigned participants to the audience condition: Half of the participants viewed these videos alone (alone condition), and the other half in the presence of two researchers (audience condition). Participants completed the first component in a fixed order (puzzle followed by performance task), after which they engaged in the viewing of these tasks (in counterbalanced order). A fixed order for the puzzle and performance tasks was used as piloting revealed that younger children who were asked to complete the performance task first were less likely to be engaged and willing to participate in the remainder of the experiment. Additionally, since we measured responses only during the viewing of these videos (which occurred after an approximately 10‐min recovery period), it was only necessary to counterbalance the presentation of the videos in this phase of the experiment.

**Recording of the puzzle solving**. Participants were instructed to solve an age‐appropriate mental‐rotation puzzle, which the experimenter claimed was very difficult. Participants performed this task in the presence of one researcher and were filmed from behind so that their face was not visible (to try to ameliorate any potential embarrassment caused by viewing one's face). Younger children were presented with a physical puzzle: a series of block patterns on pieces of paper and a collection of puzzle pieces, and were asked to place the correct puzzle piece on the example pattern in the correct orientation. The children were made to believe that the task was timed, and that they were to complete the puzzle before the experimenter's stopwatch sounded. In reality, the stopwatch never sounded, and they were free to take as much time as necessary to complete the puzzle.

Older children and adults completed the mental rotation task on a computer. They were presented with a series of monkey figures (older children) and Tetris blocks (adults): one figure was presented below a line (rotated between 45° and 225°), and two figures above a line (mirrored vertically). The objective was to mentally rotate the figure below the line, and to indicate which of the two figures above the line was the identical match. Children were given 10‐s to choose a response alternative, and completed a total of 10 trials. Adults completed a total of 20 trials, and were also given 10‐s to choose a response alternative. In both cases, participants used the “Z” key to indicate the left response alternative and the “M” key to indicate the right response alternative.

After completion of the puzzle, while being recorded, all participants received age‐appropriate false positive feedback—irrespective of actual performance in the task. Feedback was slightly adjusted between children and adults, with adults receiving a score expressed in a percentile, and children said that they scored better than all other participants. This was because piloting revealed that highly exaggerated feedback in the adult group made the task less believable, and that children had difficulty interpreting the meaning of percentages. Younger children received verbal feedback at the conclusion of the task. The experimenter faced the child and told them that they “scored better and were faster than all of the other children their age” and congratulated them. Older children received the same message, although this was read by a text‐to‐speech function after completion of the task. Adult participants received a similar text‐to‐speech message that they “scored better than 90% of participants, and were also faster than 88% of participants.” After a rest period of approximately 5‐min, participants completed the singing performance.

**Recording of singing performance**. The singing performance was identical for all age groups. Participants were asked to stand in the corner of the room and sing a song of their own choice for 1 min. The song itself was unimportant if the participant was able to stand and sing for a full minute. Participants were filmed facing the camera, such that their face and body were in full frame. In the case of the younger children, who sometimes had trouble and/or showed unwillingness to sing, the researcher cued the child on but gave no positive feedback. If the child refused to sing, the researcher offered to sing with the child. If the child still refused, the researcher offered the alternative of having the guardian stand next to the child to help sing. Finally, if the child refused this offer, the performance task ended. Out of 71 young children, 14 did not want to perform alone and therefore performed standing next to their guardian. There was no evidence that this influenced children's embarrassment. The comparison of cheek temperature change scores and nonverbal embarrassment during the viewing task between children who performed alone versus those who performed with their guardian were non‐significant, *F*(1, 58) = 2.21, *p* = 0.143, *η* ^2^ = 0.04; *F*(1, 63) = 2.23, *p* = 0.140, *η* ^2^ = 0.03, respectively. Additionally, these children were distributed across both audience conditions (*n*_alone_ = 5, *n*_audience_ = 8). Therefore, we could be confident that parental presence in the recordings did not influence our findings on embarrassment.

**Viewing task**. After recording both the puzzle and performance tasks, the researcher left the room for approximately 10 min and cut the participants’ recordings such that they were 1‐min in length. This window also gave sufficient time for participants to recover after the performance task, given that this may have resulted in a brief period of stress. Children were given the opportunity to play. After the videos were cut, the viewing task began. Both researchers returned, and participants were invited to sit in front of the viewing computer, at approximately 30 cm distance from the monitor.

Next, disposable Ag/Cl isotonic electrodes to measure SCL were placed on the intermediate phalange of both fingers on the right hand. For the placement of the ECG electrodes, we used a modified Lead II placement, with electrodes that were placed under the participant's right clavicle and on the left and right sides of the abdomen. Finally, the skin temperature probe was affixed to the participant's skin, directly under the right cheekbone with medical tape.

After the application of all electrodes, the viewing task began. The researcher turned the webcam on discreetly and hid the recording from the participants’ view. Children's parents were asked to leave the room during the viewing phase (regardless of the audience condition assigned). For participants assigned to the audience condition, both researchers sat adjacent to the participant, facing a screen which showed a live recording of their physiological response, at approximately 1‐m distance. Both the participant and the researcher could see both the recordings and the physiological response output. The researchers avoided interaction with the participants. For participants assigned to the alone condition, all individuals left the room. Participants were told that the experimenter would go to another room and would return “in a few minutes.” The viewing task began with a 1‐min baseline recording: An aquarium scene, showing various swimming fish and coral, was presented without sound, designed to be engaging for younger participants, but not too arousing as to inflate baseline measurements. After the baseline recording, participants viewed their 1‐min pride and embarrassment‐inducing videos in counterbalanced order. The video of the puzzle task was filmed from behind and depicted performing the puzzle and receiving the false positive feedback. The participant was recorded from behind to move the focus from the self and enhance the focus on the positive feedback to ensure pride would be induced (and not embarrassment due to seeing one's face). The video of the singing performance was filmed from the front and depicted the participant standing and singing a song from start to end. Between the presentation of each of the induction videos, an additional 1‐min baseline video of a similar aquarium scene was displayed (second baseline), as done in similar studies where physiological reactivity is measured to ameliorate any carry‐over effects (Brummelman et al. 2018). Viewing all the stimuli took a total of 4 min.

Finally, following completion of the video sequence, adults and older children (but not younger children) were asked to report the extent to which they felt both proud viewing their solving of the puzzle and receiving positive feedback, and embarrassed viewing their singing performance on a three‐point scale (0 = “*not at all embarrassed/proud*,” 1 = “*a little embarrassed/proud*,” 2 = “*very embarrassed/proud*”).

### 2.3 Materials

A Sony Handycam HDR‐CX240 was used to record the participants in the puzzle and performance tasks, while nonverbal emotional expressions were recorded with a Logitech C270 webcam during the viewing of these videos. A Dell Optiplex 3060 computer and a Phillips 243S monitor were used to display stimuli during both the puzzle and the viewing. The display of both videos was programmed, and event markers were sent using E‐Prime 3.0 software (Psychology Software Tools, Pittsburgh, PA). The puzzle itself was programmed using PsychoPy2 (Peirce et al. 2019).

Physiological data were recorded using AcqKnowledge v5.0 (Goleta, California) software and acquired with a BIOPAC MP150 system. An ECG100C module was used to collect ECG data, acquired with a gain of 2000 Hz, a lowpass filter of 35 Hz, and a highpass filter of 1 Hz. SCL data were collected using a GSR100C module with a gain of 5uS/V and a lowpass filter of 10 Hz. Finally, cheek temperature data were collected using a fast response thermistor (TSD202A, BIOPAC) and an SKT100C module with a gain of 2°F/V and a lowpass filter of 10 Hz.

### 2.4 Data Processing

**Physiological data**. Physiological data were pre‐processed and manually inspected using PhysioData Toolbox (Sjak‐Shie 2022). For all measurements, to ensure a continuous signal, artifacts were linearly interpolated at locations with missing and/or erroneous signal values. Raw ECG data were pre‐processed using a band‐filter with a cut‐off of 1 and 50 Hz. The toolbox manually detected R‐peaks, and each recording was manually inspected for artifacts.

We use a time‐domain index of heart rate variability (HRV)—the root mean square of successive RR interval differences (RMSSD)—to quantify the amount of variability between successive IBIs. Raw SCL data were pre‐processed using a low‐pass filter with a cut‐off of 2 Hz. No specific pre‐processing was required for cheek temperature measurements, but data were nonetheless inspected for artifacts. HRV, SCL, and cheek temperature scores were calculated over a period of 60‐s for the viewing of the performance task, and over a period of 30‐s for the viewing of the puzzle task (given that the false positive feedback was always given in the last 30‐s of the video).

In preparation for subsequent analyses, mean data were baseline corrected by calculating relative change scores. Baseline 1 was used to calculate relative change scores for the first viewing task, and Baseline 2 was used to calculate relative change scores for the second viewing task. In this way, we could measure the reaction to the viewing task relative to the period just before that task and ameliorate any carry‐over effects. Although we had originally indicated to use absolute change scores in our pre‐registration (i.e., SkinTemp_task_—SkinTemp_baseline_), this method proved unsuitable given that children and adults exhibited quite large differences in baseline physiological response. For example, adults’ cheek temperature was significantly higher than children's, which made it difficult to compare the extent of physiological change between groups using these absolute scores. Differences in (baseline) physiological reactivity between children and adults are common (Falk and Dotan 2008; Mulkey and du Plessis 2019; Porges 2009; Silvetti et al. 2001), but beyond the scope of this paper. As such, we opted for relative change scores, calculating the percentage change from baseline 1 (or 2) according to the Formula (1):

(1) $$\mathrm{Baseline}\;\mathrm{corrected}\;\mathrm{measure}\;=\left(\frac{x^{2}-x^{1}}{x^{1}}\right)\;*100,$$

where *x*_1_ is the mean of the measurement during baseline 1 and/or 2, and *x*_2_ is the mean of the measurement during the viewing of the tasks.

**Nonverbal emotional expression data**. Nonverbal emotional expression data were manually micro‐coded frame‐by‐frame in the Observer XT (Noldus, Wageningen) by a FACS‐certified coder (first author). In addition, a group of four research assistants, blind to the emotion induction condition displayed in the video, went through extensive training for micro‐coding with the first author and coded 10% of the videos to establish inter‐rater reliability. Each minute‐long video of the participant's face during the two viewing tasks was coded for seven behaviors, each with two or more mutually exclusive categories: lips corners (AU12: raised, not raised); cheek raiser (AU6: active, not active); gaze (averted, not averted); head position (AUs 53, 54, 56, 57: head tilt up, head tilt down, head tilt side); tongue protrusion (protruded, not protruded); hand position (touching face, not touching face); chest position (neutral, closed, expanded). Reliability was good (Cohen's kappa > 0.75) for all behaviors.

To derive an index of the nonverbal expression of embarrassment, the total length of time in which each participant displayed a smile (as indicated by raised lip corners) combined with averted gaze and/or a head tilt (down or to the side but not up) was summed (as per the prototypical nonverbal displays for both children and adults indicated in; Colonnesi et al. 2014; Cordaro et al. 2018; Keltner and Buswell 1997; Reddy 2004). Although, as indicated, we coded tongue protrusions and hand positions, these behaviors were fully accounted for by other behaviors, meaning their inclusion in the nonverbal expressions of embarrassment was superfluous. For example, there were no instances of participants covering their face with their hand that did not occur without a head aversion, and there were no tongue protrusions that did not occur without smiles. As such, it was superfluous to include these behaviors in our analysis. As the video duration was 60 s, the total length of the embarrassment display could vary between 0 and 60 s. To derive an index of the nonverbal expression of pride, the total length of time in which each participant displayed a smile (as indicated by raised lip corners) with direct gaze combined with a head tilt up and/or expanded chest was summed (as per the prototypical nonverbal display indicated in; Tracy and Robins 2004, 2007). Since the false positive feedback, which is assumed to evoke pride, was always displayed in the last 30‐s of the video display, we calculated the total length of the pride display (in seconds) over this period, such that it was always between 0 and 30 s.

### 2.5 Data Analysis

Main analyses were conducted using R Studio (R Studio Team 2023), and manipulation checks using JASP (JASP Team 2024). Analysis of our data in the manner we pre‐registered prevented us from explicitly examining our hypotheses—which concerned behavioral and physiological responding within each specific task. Additionally, when performing our pre‐registered analyses, we found consistently strong (and unexpected) interactions between the emotion induction task and age group on skin temperature, skin conductance level, and nonverbal expressions of embarrassment. Although we had anticipated age‐related effects for all dependent variables, we did not expect that the two viewing tasks would result in such disparate physiological and behavioral responses across age.

As such, rather than focusing on the unexpected interaction effects with the emotion induction task, which was not of interest for our study, we decided to deviate from our pre‐registered analyses and focus on each task separately. To this end, we examined whether both age group and audience condition influenced participants’ responding while viewing the videos intended to induce embarrassment and pride in separate models for each emotion. Nonetheless, for transparency, we present our pre‐registered analyses in Supporting Information 1. We inspected all variables for outliers before running our models, and, where possible, winsorized values greater or less than three standard deviations from the mean, preserving rank order.

To this end, for both viewing tasks separately, we performed a series of two‐way ANOVAs with audience condition and age group as fixed factors and participants’ skin temperature, skin conductance level, nonverbal expressions of embarrassment, pride, and self‐reported embarrassment/pride as outcome measures. Since the assumption of normality for ANOVA was seriously violated for the self‐report data, we performed a series of non‐parametric Kruskal‐Wallis tests on these data, with age group and audience condition as fixed factors and participants’ self‐report scores as the outcome measure. In the event of significant effects, we followed up these tests with Dunn's tests for pairwise comparisons across factor levels. A further exception to this analysis strategy was for the pride nonverbal expression data, for which we used the glmmTMB function (Brooks et al. 2017) to model zero‐inflated Gamma distributions with a log link, as nonverbal pride expression duration scores were highly positively skewed (Mahmood and Xie 2019), with a large number of zeros. Note that since this model considers the participants who displayed pride while watching the puzzle task (*n* = 46) separately from those who did not (*n* = 170), it is based on fewer observations than the models above.

### 2.6 Transparency and Openness

We report how we determined our sample size, all data exclusions (if any), all manipulations, and all measures in the study, and the study follows JARS (Appelbaum et al. 2018). All non‐identifiable data, analysis code, and research materials are available at: https://osf.io/ac3sv/.

## 3 Results

Descriptive statistics of all self‐report, nonverbal expression, and physiological variables for all age groups, audience conditions, and emotion induction tasks can be found in Tables S1–S6 of Supporting Information 2. A correlation matrix of the dependent measures can be found in Supporting Information 3.

### 3.1 Manipulation Check

To begin, we examined whether our manipulations resulted in the expected patterns of emotional responding within and across tasks. In terms of physiological activity, we examined whether the viewing of the singing and puzzle tasks resulted in the hypothesized autonomic arousal response by comparing participants’ responding in each viewing task compared to baseline using a series of paired‐sample *t*‐tests. As expected, participants’ skin conductance level scores were significantly greater and heart rate variability scores significantly lower than baseline when viewing the puzzle task, *t*(209) = 3.25, *p* = 0.001, *d* = 0.22; *t*(207) = –2.12, *p* = 0.036, *d* = –0.15, respectively. When viewing the singing task, we found a similar pattern of autonomic activity. Participants’ skin conductance level scores increased significantly from baseline, *t*(209) = 11.17, *p* < 0.001, *d* = 0.77, while participants’ heart rate variability decreased significantly from baseline, *t*(207) = –3.50, *p* < 0.001, *d* = –0.24. This pattern of responding suggests that we did induce the expected pattern of emotional arousal during both viewing tasks. Finally, participants’ cheek temperature during the viewing of the puzzle, *t*(202) = 3.99, *p* < 0.001, *d* = 0.28, and performance viewings, *t*(202) = 7.42, *p *< 0.001, *d* = 0.52, were significantly higher than baseline levels. This indicates that both tasks also induced specific self‐conscious physiological arousal.

Next, we examined participants’ nonverbal expressions of pride and embarrassment across both viewing tasks. First, we checked whether participants expressed greater than zero levels of nonverbal pride and embarrassment expressions in each task separately. During the viewing of the puzzle task, participants expressed nonverbal pride and embarrassment significantly above zero levels, *t*(215) = 3.75, *p* < 0.001, *d* = 0.26; *t*(209) = 9.56, *p *< 0.001, *d* = 0.66. Similarly, during the viewing of the singing task, participants expressed nonverbal pride and embarrassment behaviors significantly above zero levels, *t*(215) = 5.03, *p* < 0.001, *d* = 0.34; *t*(208) = 15.94, *p* < 0.001, *d* = 1.11, respectively. This suggests that, contrary to our expectations, both tasks resulted in the production of both nonverbal expressions of emotion.

Next, and more critically, we compared nonverbal embarrassment and pride expressions within each viewing task separately using a series of chi‐squared tests to examine whether the tasks evoked one specific self‐conscious emotion, rather than more general self‐conscious emotional arousal. For these direct comparisons between embarrassment and pride, we looked at the presence and absence of each nonverbal expression within each task. This was because nonverbal expressions of pride were typically more transient in duration (i.e., shorter) than those of embarrassment and were related specifically to the presentation of the positive feedback (which always occurred for a short period in the last 30 s of the video). Nonverbal expressions of embarrassment, rather, were more likely to be evoked and displayed for the duration of both tasks. During the viewing of the puzzle, participants did not express more nonverbal expressions of pride than embarrassment, χ^2^(1) = 0.05, *p* = 0.816. During the viewing of the singing task, participants expressed significantly more nonverbal expressions of embarrassment than pride, χ^2^(1) = 18.10, *p *< 0.001. Full model summaries for each of the manipulation checks can be found in Supporting Information 4.

To summarize, this pattern of results suggests that the viewing of the performance task was successful in inducing embarrassment specifically, but the viewing of the puzzle task induced both pride *and* embarrassment. Nonetheless, since we still did induce nonverbal expressions of pride, we decided to proceed with our analyses as originally planned. Because we induced both self‐conscious emotions in the viewing of the puzzle task, we analyzed and reported the models with both embarrassment and pride nonverbal expressions in both viewing tasks (to show how they differed across age and audience conditions).

### 3.2 Did Audience Presence Influence Emotional Responding During the Viewing Tasks?

**FIGURE 1:**
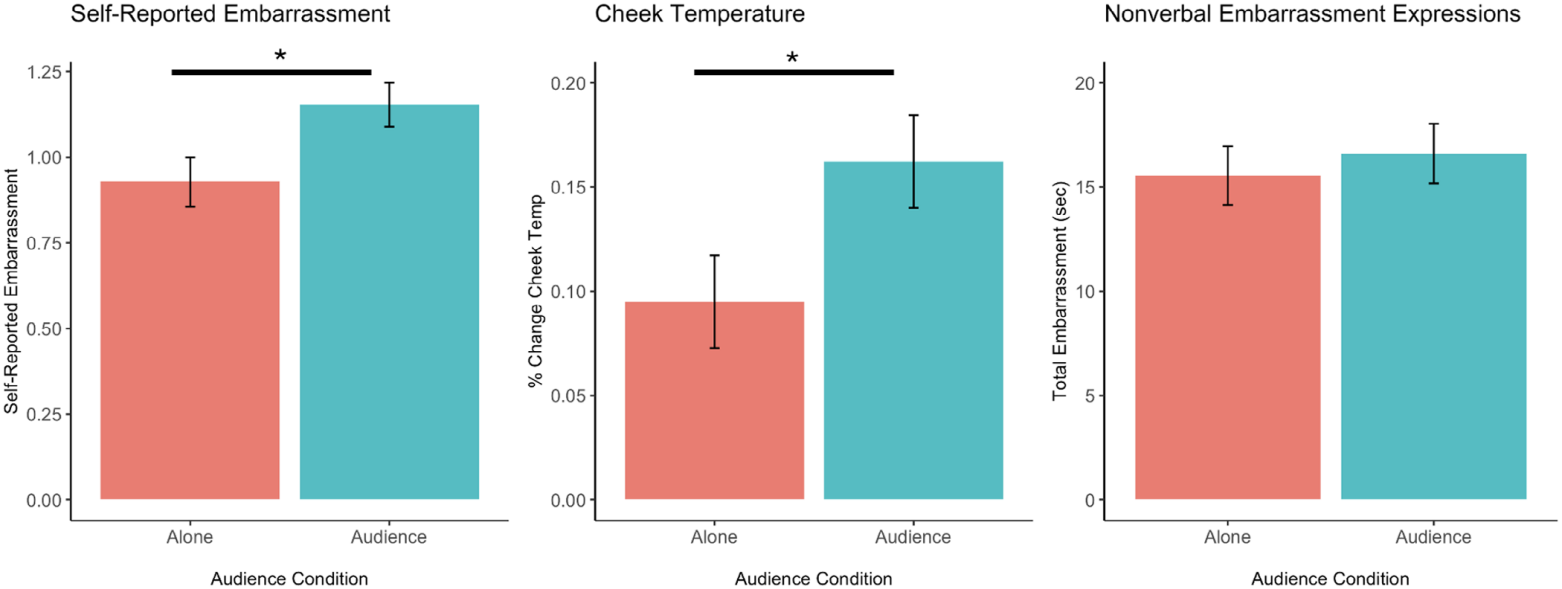
Effect of audience presence on participants’ self‐reported embarrassment, nonverbal expressions of embarrassment, and cheek temperature during the viewing of the singing performance. *p < 0.05. Error bars represent standard error.

**Singing performance**. While viewing themselves sing, we found a significant main effect of audience presence on participants’ cheek temperature change scores, *F*(1, 197) = 4.56, *p* = 0.034, η_p_^2^ = 0.02 (Table 1). This effect was such that, as hypothesized, participants showed greater increases in cheek temperature in the presence of the audience than when alone, *M*_difference_ = 0.07, *p* = 0.034 (Figure 1). Additionally, we found a significant audience effect on older children and adults’ self‐reported embarrassment in the same direction, *χ^2^* (1) = 5.17, *p* = 0.023, with participants reporting more embarrassment in the presence of the audience than when alone while viewing themselves perform (*Z* = 2.23) (Figure 1). No other significant audience effects on emotional responding could be found across our other response measures (nonverbal expressions of embarrassment, nonverbal expressions of pride, skin conductance level, and heart rate variability) when participants viewed themselves singing, all *p*’s > 0.05 (Tables 1 and 4).

**TABLE 1:** Results of a series of two‐way ANOVAs predicting cheek temperature, skin conductance level, heart rate variability, and nonverbal expressions of embarrassment scores by age and audience condition during the viewing of the singing performance.

	Cheek temperature				Skin conductance level				Heart rate variability				Nonverbal embarrassment			
Predictor	*df*	*F*	*p*	η_p_^2^	*df*	*F*	*p*	η_p_^2^	*df*	*F*	*p*	η_p_^2^	*df*	*F*	*p*	η_p_^2^
Age Group	2	2.82	0.062	0.03	2	0.77	0.467	0.01	2	2.58	0.079	0.02	2	7.72	<0.001	0.07
Condition	1	4.56	0.034	0.02	1	0.24	0.628	0.00	1	0.48	0.488	0.00	1	0.37	0.543	0.00
Age Group * Condition	2	1.51	0.224	0.02	2	1.47	0.232	0.01	2	0.56	0.573	0.01	2	0.75	0.475	0.01
Residuals	197				204				202				203			

**Puzzle task**. While viewing themselves completing the puzzle and receiving positive feedback, we found no audience effects across all our response measures (nonverbal expressions of embarrassment, nonverbal expressions of pride, cheek temperature, skin conductance level, heart rate variability, and subjectively reported pride), all *p*’s > 0.05 (Tables 2 and 3). This suggests that audience presence did not influence self‐conscious emotions during the viewing of the puzzle task.

**TABLE 2:** Results of a series of two‐way ANOVAs predicting cheek temperature, skin conductance level, and heart rate variability and nonverbal expression of embarrassment scores by age and audience condition during the viewing of the positive feedback.

	Cheek temperature				Skin conductance level				Heart rate variability				Nonverbal embarrassment			
Predictor	*df*	*F*	*p*	η_p_^2^	*df*	*F*	*p*	η_p_^2^	*df*	*F*	*p*	η_p_^2^	*df*	*F*	*p*	η_p_^2^
Age Group	2	9.06	<0.001	0.08	2	15.22	<0.001	0.13	2	6.68	0.002	0.06	2	5.78	0.004	0.05
Condition	1	0.34	0.561	0.00	1	0.32	0.572	0.00	1	1.20	0.276	0.01	1	0.41	0.521	0.00
Age Group * Condition	2	1.05	0.351	0.01	2	0.97	0.383	0.01	2	0.60	0.553	0.01	2	2.24	0.109	0.02
Residuals	197				204				202				204			

**TABLE 3:** Results of zero‐inflated gamma generalized linear model predicting pride nonverbal expression duration by age group, emotion, and audience during the viewing of the positive feedback.

	Total pride		
Predictors	Estimates	CI	*p*
Count model			
(Intercept)	8.85	2.67–29.28	<0.001
Audience condition			
*Audience (Reference)*			
*Alone*	0.62	0.15–2.58	0.509
Age category			
*Adult (Reference)*			
*Older Child*	0.29	0.07–1.11	0.071
*Younger Child*	0.15	0.04–0.64	0.010
Audience Condition * Age Category			
Alone * Older Child	1.97	0.36–10.72	0.432
Alone * Younger Child	3.43	0.57–20.49	0.177
(Intercept)	2.88	2.42–3.54	
Zero‐Inflated Model			
(Intercept)	3.70	2.67–5.12	<0.001
Observations	216		
R^2^ marginal	0.267		

### 3.3 Were Their Age‐Related Differences in Emotional Responding During the Viewing Task?

**FIGURE 2:**
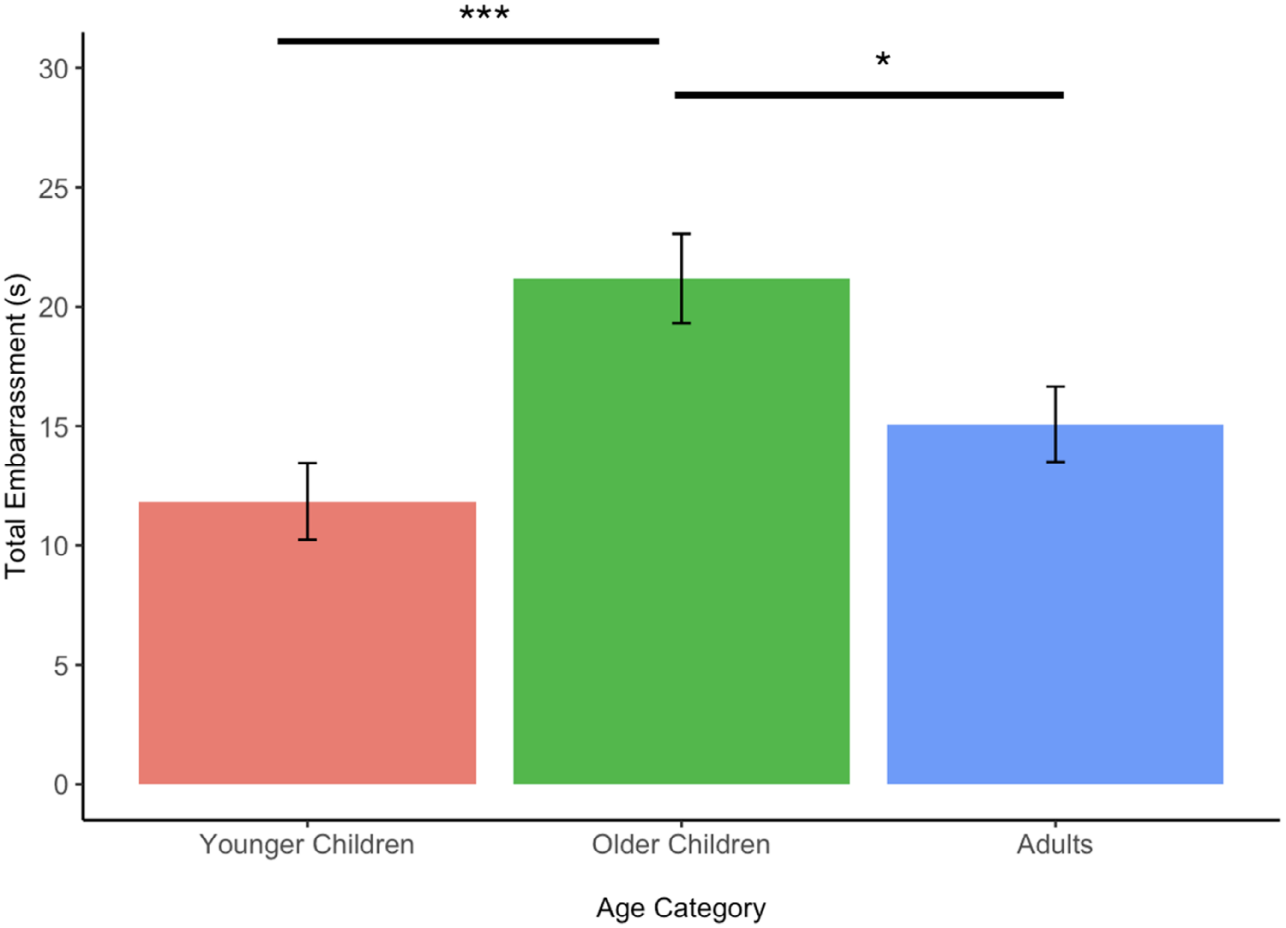
Bar plots indicating total displayed nonverbal expressions of embarrassment during the viewing of the singing performance per age group. * *p* < 0.05, ***p < .001. Error bars represent standard error.

**Singing performance**. While participants viewed themselves sing, we found no age‐related differences in participants’ cheek temperature and skin conductance level (Table 1), as well as nonverbal expressions of pride (Table 4), all *p*’s > 0.05. However, there were significant differences in the length of nonverbal expressions of embarrassment displayed across age groups, *F*(2, 203) = 7.71, *p *< 0.001, η_p_^2^ = 0.07 (Table 1). Older children displayed significantly more nonverbal expressions of embarrassment compared to both younger children and adults, *M_difference_* = 9.32, *p *< 0.001, *M_difference_* = 6.09, *p* = 0.032, respectively. The difference between adults and younger children was not significant, *p *= 0.537. These age‐related differences in nonverbal expressions of embarrassment are displayed in Figure 2. In terms of subjectively reported embarrassment, Kruskal‐Wallis tests revealed no differences in self‐reported embarrassment between age groups in this task, *χ^2^* (1) = 0.08, *p *= 0.779.

**TABLE 4:** Results of zero‐inflated gamma generalized linear model predicting pride nonverbal expression duration by age group, emotion, and audience during the viewing of the singing task.

	Total pride		
Predictors	Estimates	CI	*p*
Count model			
(Intercept)	3.45	1.69–7.06	**0.001**
Audience condition			
*Audience (Reference)*			
*Alone*	0.64	0.25–1.66	0.359
Age category			
*Adult (Reference)*			
*Older Child*	0.80	0.24–2.61	0.711
*Younger Child*	0.73	0.28–1.88	0.509
Audience Condition * Age Category			
Alone * Older Child	2.04	0.47–8.87	0.342
Alone * Younger Child	1.18	0.32–4.35	0.803
Zero‐inflated model			
(Intercept)	3.50	2.54–4.82	**<0.001**
Observations	216		
R^2^ Nagelkerke	0.019		

**FIGURE 3:**
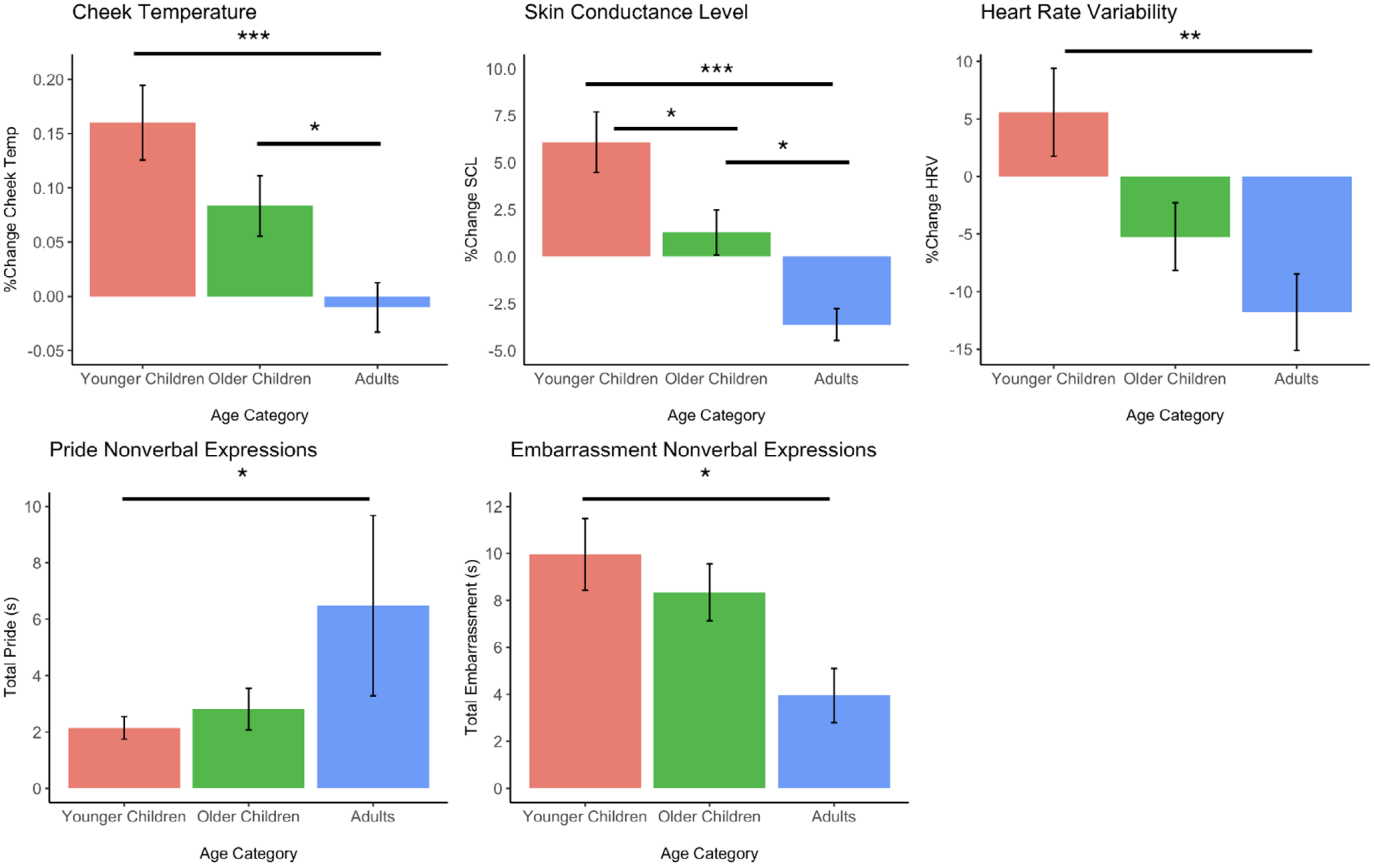
Effect of age group on participants' cheek temperature, skin conductance level, heart rate variability, nonverbal expressions of pride, and nonverbal expressions of embarrassment during the viewing of the positive feedback. Effects of age group on pride nonverbal expressions based on a zero‐inflated generalized linear model, and includes only the participants who displayed at least some pride nonverbal behaviors (*n =* 46 individuals). *p < .05, **p < .01, *** *p* <.001.

**Puzzle task**. While participants viewed themselves receiving positive performance feedback, we found a significant effect of age group on cheek temperature change scores, *F*(2, 197) = 9.06, *p* < 0.001, η_p_^2^ = 0.08 (Table 2). Both younger and older children's cheek temperature rose significantly more than adults, *M_difference_* = 0.17, *p *< 0.001; *M_difference_* = 0.09, *p *= 0.049 (Figure 3), respectively, indicating that young children showed stronger self‐conscious emotional arousal compared to adults when viewing themselves solve the puzzle and receiving a compliment for this. There were also significant differences across age groups on skin conductance level change scores, *F*(2, 204) = 15.22, *p *< 0.001, η_p_^2^ = 0.13 (Table 2), with both younger and older children's skin conductance level rising significantly more than that of adults, *M_difference_* = 9.70, *p *< 0.001, *M_difference_* = 4.89, *p* = 0.016, respectively. Younger children also demonstrated a greater positive skin conductance level change compared to older children, *M_difference_* = 4.81, *p* = 0.022 (Figure 3). In terms of the changes in heart rate variability scores during this task, we found significant effect of age group, *F*(2, 202) = 6.68, *p *= 0.002, η_p_^2^ = 0.06, with younger children heart rate variability rising significantly more than that of adults, *M_difference_* = 17.36, *p *= 0.001. Together, these findings indicate a stronger general emotional arousal in children (and younger children specifically) compared to adults.

Finally, analysis of nonverbal expressions of pride also revealed a significant effect of age category, *β* = –1.88, *p *= 0.010, 95% CI = [0.04–0.64]. Taking the exponent of this coefficient revealed that the expected nonverbal displays of pride for younger children were approximately 15% less than the expected nonverbal displays of pride for adults (the reference category; Table 3, Figure 3). Additionally, during the viewing of the puzzle task, we found a significant effect of age group on participants’ nonverbal expressions of embarrassment, *F*(2, 204) = 5.78, *p *= 0.004, η_p_^2^ = 0.05, with younger children displaying significantly more embarrassment compared to adults, *M_difference_* = 5.92, *p *= 0.004 (Figure 3). These results indicate that viewing the puzzle task evoked less pride and more embarrassment in young children compared to adults. Finally, in terms of self‐reported pride, there was also a significant effect of age group, *χ^2^* (1) = 43.71, *p *< 0.001, with Dunn post‐hoc tests revealing older children reported significantly more pride compared to adults after viewing themselves receiving praise for their success in the puzzle (*Z* = 6.61).

## 4 Discussion

Emotions, and in particular emotional expressions, do not exist in isolation, but rather appear within, and are highly influenced by, the social environment. Audience effects provide an interesting avenue for prying into how the self‐reported experience and expression of emotions are influenced by social context, and have therefore been the focus of much scholarly work (Fridlund 1991; Fridlund et al. 1992; Hamilton and Lind 2016; Holodynski 2004; Holodynski 2006). Additionally, the examination of when and in which contexts sensitivity to audience presence emerges provides critical insights into children's burgeoning self‐representation and impression management skills (Botto and Rochat 2019). In this pre‐registered experiment, we examined whether being in the company of others modulates emotional responding for two specific positive and negative self‐conscious emotions—pride and embarrassment—and whether this differs across various stages of human development.

We found that participants reported feeling more embarrassed while watching themselves perform when they were in the presence of others compared to when they were alone. In line with this, participants also showed greater increases in cheek temperature in the audience condition, reflecting more physiological blushing (Schandry and Poth 1983). Interestingly, although reporting feeling more embarrassed and blushing more, participants did not show more nonverbal expressions of embarrassment in the presence of others. Emotional responding when participants watched themselves receive positive feedback; however, was not influenced by the presence of the audience. Rather, we found clear age‐related differences: children experienced more physiological arousal and reported experiencing more pride than adults. However, when looking at their nonverbal expressions of pride during this task, we found the opposite pattern: adults expressed more nonverbal pride behaviors than children.

These findings have important implications for theories on how emotions and emotional expressions develop and are influenced by social context (Fischer and Hess 2017; Holodynski 2004; van Kleef et al. 2016), and for social cognition more broadly (Kampis and Southgate 2020). Importantly, our results may suggest a potential distinction between automatic emotional cues and those that are under more volitional control.

### 4.1 Audience Effects on Emotional Responding

Despite having hypothesized that being in the presence of an audience would amplify emotional responding more generally, we found this to be only partly true. To this end, participants’ cheek temperature increased in the presence of others only when they viewed their singing performance (but not when they viewed themselves receiving positive feedback). Cheek temperature increases are assumed to reflect physiological blushing (Schandry and Poth 1983; Shearn et al. 1990), and blushing is known to be a hallmark physiological response specific to self‐conscious emotional arousal, including both negative and positive social attention (Crozier 2004, 2007; Leary et al. 1992). Similarly, adults and older children also reported experiencing more embarrassment while watching back their singing performance in the presence of others, indicating that the presence of others increased feelings of embarrassment. This result is in line with the idea that embarrassment, and blushing in particular, is elicited by feelings of being exposed to others’ attention, being scrutinized, and judged (Crozier 2004, 2007; Leary et al. 1992).

It is important to note that although the effect size for the effect of audience on physiological blushing was small‐to‐medium, it was nonetheless consistent with older children and adults’ self‐reports of experiencing greater feelings of embarrassment while watching their singing performance in the presence of others compared to when they were alone. This suggests that the presence of others evoked embarrassment in this task, both on a subjective and physiological level. Interestingly, we did not find evidence for differences in other physiological measures than cheek temperature across audience conditions. Unlike blushing, which is specific to self‐conscious emotions (Leary and Meadows 1991), other physiological measures (skin conductance and heart rate variability) are reflective of a more general arousal (Kreibig 2010; Porges 2009) and may appear with many different social and non‐social emotions. This may explain why the presence of an audience did not have a clear effect on these measures.

Although participants felt more embarrassed and showed greater increases in cheek temperature in the audience condition, this was not reflected in their nonverbal emotional expressions. Nonverbal expressions of emotions, like changes in cheek temperature, are directly visible to observers, and we had hypothesized that audience presence would similarly amplify these displays. As such, it is interesting that, amongst both the nonverbal expression and cheek temperature domains (both of which are visible to outside observers), we found an audience effect for cheek temperature only. The lack of audience effects on participants’ nonverbal behavior does support one previous study, which found that children expressed similar levels of shame when they were observed by others during a failure to help compared to when they were not (Gerdemann, Tippmann, et al. 2022). It is, therefore, possible that participants in the audience condition attempted to downregulate their behavior in order to appear less embarrassed than they actually were (Costa et al. 2001). One's cheek temperature (and other responses of the autonomic nervous system) is largely uncontrollable and, therefore, less susceptible to regulation. Conversely, nonverbal expressions of emotion can be somewhat controlled (Kromm et al. 2015; Recio and Sommer 2018).

Although often resulting in positive social outcomes for the individual displaying the embarrassment (Feinberg et al. 2012), embarrassment itself is seen as a negative and aversive emotional state (Miller 1995), and people often try to avoid or hide it (Crozier 2007; Dong et al. 2013). This may explain why we found an effect of audience presence in the skin temperature domain only. This result is in line with one previous study (Costa et al. 2001) that found that although participants reported feeling more embarrassment in the presence of an audience compared to when they were alone, they demonstrated significantly less nonverbal embarrassment behaviors in the audience condition. The authors argue that this may have reflected participants’ use of emotion regulation strategies in order to try to appear less embarrassed than they actually were. This may explain why we did not find any effects of audience presence on participants’ nonverbal expressions of embarrassment.

Moreover, although we hypothesized that the effects of the audience would be largest for adults, we found that the positive effect of the audience on cheek temperature was consistent across all age groups during the viewing of the singing performance. Since pride and embarrassment (as well as other social emotions) have been argued to have an attenuated development across childhood compared to other emotions (Lewis et al. 1989; Somerville et al. 2013), we expected that older children and adults would be more sensitive to the presence of the audience than younger children, and this would be reflected in greater increases in cheek temperature. However, cheek temperature increases across audience conditions did not differ between age groups, which may indicate that watching oneself perform induced physiological blushing—a hallmark of embarrassment (Buss 1980)—consistently across our sample of children and adults. To our knowledge, no studies have directly examined both children's and adults’ cheek temperature changes during a contextually similar embarrassment‐inducing task. As such, this study represents one of the first pieces of evidence that young children as young as three years old can blush in a similar manner to older children and adults, at least in the domain of embarrassment. This suggests that very young children may be, like older children and adults, sensitive to social exposure and possible negative evaluations by others.

We observed significant age‐related differences in nonverbal expressions of embarrassment—with older children displaying significantly more embarrassment expressions while watching their signing performance compared to both younger children and adults. Although we hypothesized that adults would display the most nonverbal expressions of embarrassment due to their further‐developed socio‐cognitive skills, this result nonetheless supports previous works on the development of social emotions across ontogeny. For example, in one study, during a task in which participants believed they were being observed by a peer, participants’ self‐reported embarrassment and autonomic arousal were found to be highest in adolescence, tapering off with the emergence of adulthood (Sommerville et al. 2013). Our results point to a similar pattern with children approaching pre‐adolescence displaying the most embarrassment of all age groups. Considering that older children, when compared to adults, did not differ in self‐reported embarrassment and physiological blushing, this result may indicate that they were less able to downregulate their nonverbal expressions of embarrassment whilst viewing their singing performance.

Our results beg the interesting question of why, contrary to our predictions, we did not find any positive effect of audience presence on participants’ cheek temperature when they viewed themselves receiving a compliment for solving a puzzle well. Considering that blushing is also assumed to occur with positive attention (Leary et al. 1992), we expected to see participants blush more while viewing themselves receive compliments in the presence of others compared to when they were alone. However, this was not the case. A simple explanation could be that being in the presence of the audience did not induce more self‐conscious emotional arousal compared to being alone. In line with this, we did not find similar audience effects across other physiological measures, nonverbal behavior, and self‐report scores when participants watched themselves receive praise. Conversely, we found clear age‐related differences in physiological responding and nonverbal behavior during this task. Compared to adults, across both cheek temperature and skin conductance measures, both older and younger children demonstrated higher physiological arousal. Thus, it seems that receiving a compliment for solving a puzzle well is especially arousing in childhood. In line with this, older children also reported experiencing significantly more pride after viewing themselves receiving positive feedback compared to adults. Interestingly, adults displayed the most nonverbal expressions of pride, although this result should be interpreted with caution given the low number of pride observations used in the modelling of these data.

Although we attempted to mitigate this with our design, the task intended to induce pride also appeared to have the unintended effect of inducing embarrassment, perhaps mostly for the younger children, who displayed significantly more embarrassment and less pride than adults. We found some nonverbal expressions of embarrassment (in addition to pride) in the two child age groups (although the duration of these expressions was significantly lower than during the viewing of the singing performance; see Supporting Information 1, Table S7). Indeed, there is evidence that (exaggerated) complimenting may elicit embarrassment in late childhood (Lewis et al. 1989, Lewis et al. 1991; Nikolić et al. 2018). Moreover, simply observing oneself may also elicit feelings of exposure and heightened awareness (Lewis and Ramsay 2002), and this might have been particularly marked for children who have had less experience viewing themselves on film compared to adults. In general, these results imply that watching oneself receive compliments for solving a puzzle well may evoke heightened self‐awareness, especially in children, and that watching oneself back on a video in a positive light can induce pride in combination with other self‐conscious emotions, such as embarrassment. As such, children may become highly self‐aware from an early age, although their socio‐cognitive skills still undergo significant development.

Although offering several important insights into the development of social emotions, as well as the impact of the audience on the expression and self‐reported experience of these across ontogeny, there are some limitations of our design worth mentioning. First, most of our participants were White and from a Western country. As such, we cannot generalize our results beyond this specific context to different populations. Moreover, although the combination of both physiological responses and self‐reports suggests that participants who viewed themselves as receiving positive feedback experienced pride, we only observed its associated nonverbal expression in one quarter of the total observations. Based on the results of our manipulation check, the puzzle task elicited embarrassment in addition to pride (i.e., mixed emotions), particularly since we measured emotional responding during the viewing phase (and not in‐the‐moment). False positive feedback tasks have been used in previous studies on pride (Adams et al. 2020; Stoeber et al. 2007; Williams and DeSteno 2008); however, future studies may consider a stronger and in‐the‐moment elicitation method in order to induce more nonverbal expressions of pride. Nonetheless, given that we still did see participants express pride (nonverbally and via self‐report), we believe our data still provide valuable insights into the ontogeny of both embarrassment and pride (and their modulation by audience).

An additional limitation of this study concerns our lack of self‐report data for younger children. Pilot testing revealed that younger children had difficulty reliably reporting on self‐conscious emotions. It may be fruitful for future studies to implement more readily interpretable scale items (e.g., emoticons rather than verbal categories) so that the subjective experience of embarrassment and pride can be properly characterized in children of this age. Moreover, it is important to note that we considered only the prototypical nonverbal displays of embarrassment (Colonnesi et al. 2014; Keltner and Buswell 1997; Reddy 2004) and pride (Tracy and Robins 2004, 2007) in our analyses. Some scholars (e.g., Barrett et al. 2019; Durán and Fernández‐Dols 2021; Russell and Barrett 1999) have questioned the extent to which such potentially stereotyped displays reflect the ways in which emotions are expressed in real‐life contexts, potentially limiting the generalizability of our findings. Although a review of this debate is beyond the scope of the present study, we believe that the addition of both physiological and self‐report measures provides nonetheless a more holistic assessment of the ontogeny of these two emotions. Furthermore, our study only investigated two self‐conscious emotions (one positive, one negative), but the range of self‐conscious emotions is wider. For example, there is debate regarding whether or not shame expressions in children may be similarly pervious to audience presence (Gerdemann, McAuliffe, et al. 2022; Holodynski 2006). Particularly, one study found that children in late childhood were more likely to express negative bodily expressions (e.g., slouched shoulders indicating shame or guilt) after receiving an advantageously unfair payout compared to a peer, but only in a social context (Gerdemann, McAuliffe, et al. 2022). This would indicate that the presence of an audience in situations that evoke shame (and perhaps other self‐conscious emotions, too) may result in the amplification of their expression, although future works should consider examining a wider range of self‐conscious emotions.

Furthermore, in our study, the audience consisted of unfamiliar strangers. Therefore, a fruitful avenue for future research may be to manipulate the composition of the audience and examine whether children and adults experience and express self‐conscious emotions differently depending on who is observing them. Importantly, previous studies have emphasized differences in emotional responding depending on the composition of the audience (e.g., friends vs. strangers, peers vs. parents). These past studies found that individuals typically tend to suppress their emotional expressions in the presence of strangers compared to friends (Manokara et al. 2023; Jakobs et al. 1999), even in childhood (Zeman and Garber 1996). The tendency to suppress appears to be stronger for negative as compared to positive emotions (Matsumoto et al. 2008). It may be expected that the type of audience influences self‐conscious emotional experience and expression in a similar manner. As such, our current findings may be amplified in the context of interactions with familiar others.

Ultimately, the data from this study paint an interesting picture regarding the nature of embarrassment and pride across both the lifespan and across social context. One key advantage of our paradigm is in its ability to capture emotional responding across modalities (e.g., physiological, behavioral, and self‐report) and to examine how each of these may be uniquely impacted by age and the presence of audience. Many common theories of emotion posit that there should be coherence between these modalities (Mauss et al. 2005). In our study, we find limited evidence of this and highlight that both age and audience presence may influence the emotional responses associated with pride and embarrassment. Concerning audience effects, visible physiological cues, such as changes in cheek temperature reflecting physiological blushing, may be particularly insightful as they are under limited volitional control and directly visible to outside observers. In terms of age, our findings suggest that children may experience and display self‐conscious emotions at least as much as adults and may, therefore, be sensitive to others around them, even at an early age. These findings, therefore, offer important insights both for theories of emotion and socio‐cognitive development more generally. Regarding the former, our results highlight the highly social nature of self‐conscious emotions and show that different modalities may not be equally influenced by the social context. Regarding the latter, our findings suggest that self‐presentation concerns may appear earlier in ontogeny than previously thought.

## Author Contributions

**Christopher Riddell**: conceptualization, methodology, formal analysis, writing (original draft), visualization. **Milica Nikolic**: conceptualization, methodology, writing – review and editing, supervision. **Mariska Kret**: conceptualization, methodology, writing – review and editing, funding acquisition.

## Ethics Statement

The study was approved by the Leiden University Ethical Review Board (Kret‐V3‐3521) and conducted in accordance with the Declaration of Helsinki. All adult participants and the parents of all child participants provided informed consent before beginning the experiment.

## Conflicts of Interest

The authors declare no conflicts of interest.

## Supporting information



Supporting information

Supporting Information

Supporting Information

Supporting Information

## Data Availability

All non‐identifiable data, analysis code, and research materials are available at: https://osf.io/ac3sv/. The hypotheses of this study were pre‐registered on the Open Science Framework: https://osf.io/rkphq.

## References

[desc70024-bib-0001] Adams, I. , K. Hurst , and N. D. Sintov . 2020. “Experienced Guilt, but Not Pride, Mediates the Effect of Feedback on Pro‐environmental Behavior.” Journal of Environmental Psychology 71: 101476. 10.1016/j.jenvp.2020.101476.

[desc70024-bib-0112] Appelbaum, M. , H. Cooper , R. B. Kline , E. Mayo‐Wilson , A. M. Nezu , and S. M. Rao . 2018. “Journal Article Reporting Standards for Quantitative Research in Psychology: The APA Publications and Communications Board Task Force Report.” American Psychologist 73, no. 1: 3. 29345484 10.1037/amp0000191

[desc70024-bib-0002] Asaba, M. , and H. Gweon . 2022. “Young Children Infer and Manage What Others Think About Them.” Proceedings of the National Academy of Sciences 119, no. 32: e2105642119. 10.1073/pnas.2105642119. PMC9371656 35930665

[desc70024-bib-0003] Banerjee, R. 2002. “Audience Effects on Self‐Presentation in Childhood.” Social Development 11, no. 4: 487–507. 10.1111/1467-9507.00212.

[desc70024-bib-0004] Barrett, L. F. , R. Adolphs , S. Marsella , A. M. Martinez , and S. D. Pollak . 2019. “Emotional Expressions Reconsidered: Challenges to Inferring Emotion From Human Facial Movements.” Psychological Science in the Public Interest 20, no. 1: 1–68. 10.1177/1529100619832930. 31313636 PMC6640856

[desc70024-bib-0005] Baumeister, R. F. 1982. “A Self‐Presentational View of Social Phenomena.” Psychological Bulletin 91, no. 1: 3.

[desc70024-bib-0006] Behnke, M. , S. D. Kreibig , L. D. Kaczmarek , M. Assink , and J. J. Gross . 2022. “Autonomic Nervous System Activity During Positive Emotions: A Meta‐Analytic Review.” Emotion Review 14, no. 2: 132–160. 10.1177/17540739211073084.

[desc70024-bib-0007] Bond, C. F. 1982. “Social Facilitation: A Self‐Presentational View.” Journal of Personality and Social Psychology 42, no. 6: 1042–1050. 10.1037/0022-3514.42.6.1042.

[desc70024-bib-0008] Botto, S. V. , and P. Rochat . 2019. “Evaluative Audience Perception (EAP): How Children Come to Care About Reputation.” Child Development Perspectives 13, no. 3: 180–185. 10.1111/cdep.12335.

[desc70024-bib-0009] Bradley, M. M. , and P. J. Lang . 2007. “Emotion and Motivation.” In Handbook of Psychophysiology, 3rd ed, 581–607. Cambridge University Press. 10.1017/CBO9780511546396.025.

[desc70024-bib-0010] Brooks, M. E. , K. Kristensen , K. J. Van Benthem , et al. 2017. “glmmTMB Balances Speed and Flexibility Among Packages for Zero‐Inflated Generalized Linear Mixed Modeling.” R Journal 9, no. 2: 378–400.

[desc70024-bib-0011] Brummelman, E. , M. Nikolić , and S. M. Bögels . 2018. “What's in a Blush? Physiological Blushing Reveals Narcissistic Children's Social‐Evaluative Concerns.” Psychophysiology 55, no. 10: e13201. 10.1111/psyp.13201. 29876926

[desc70024-bib-0012] Buss, A. H. 1980. Self‐Consciousness and Social Anxiety. WH Freeman.

[desc70024-bib-0013] Caldwell, A. , D. Lakens , L. DeBruine , J. Love , and F. Aust . 2022. Superpower: Simulation‐Based Power Analysis for Factorial Designs (Version 0.2.0) [Computer Software]. https://CRAN.R‐project.org/package=Superpower.

[desc70024-bib-0014] Citing RStudio . 2023. Posit Support. https://support.posit.co/hc/en‐us/articles/206212048‐Citing‐RStudio.

[desc70024-bib-0015] Colonnesi, C. , E. Napoleone , and S. M. Bögels . 2014. “Positive and Negative Expressions of Shyness in Toddlers: Are They Related to Anxiety in the Same Way?” Journal of Personality and Social Psychology 106, no. 4: 624–637. 10.1037/a0035561. 24564372

[desc70024-bib-0016] Cordaro, D. T. , R. Sun , D. Keltner , S. Kamble , N. Huddar , and G. McNeil . 2018. “Universals and Cultural Variations in 22 Emotional Expressions Across Five Cultures.” Emotion (Washington, D.C.) 18, no. 1: 75–93. 10.1037/emo0000302. 28604039

[desc70024-bib-0017] Costa, M. , W. Dinsbach , A. S. R. Manstead , and P. E. R. Bitti . 2001. “Social Presence, Embarrassment, and Nonverbal Behavior.” Journal of Nonverbal Behavior 25, no. 4: 225–240. 10.1023/A:1012544204986.

[desc70024-bib-0018] Crozier, W. R. 2004. “Self‐Consciousness, Exposure, and the Blush.” Journal for the Theory of Social Behaviour 34, no. 1: 1–17.

[desc70024-bib-0019] Crozier, W. R. 2007. “In Praise of Blushing.” Journal of Cosmetic Dermatology 6, no. 1: 68–71. 10.1111/j.1473-2165.2007.00292.x. 17348999

[desc70024-bib-0020] Crozier, W. R. , and P. J. de Jong . 2012. The Psychological Significance of the Blush. Cambridge University Press.

[desc70024-bib-0021] Dawson, M. E. , A. M. Schell , and D. L. Filion . 2007. “The Electrodermal System.” In Handbook of Psychophysiology, 3rd ed, 159–181. Cambridge University Press. 10.1017/CBO9780511546396.007.

[desc70024-bib-0022] de Vente, W. , M. Majdandžić , and S. Bögels . 2014. “The Pathophysiology of Social Anxiety.” In The Wiley Blackwell Handbook of Social Anxiety Disorder, 90–110. Wiley Blackwell. 10.1002/9781118653920.ch5.

[desc70024-bib-0023] Dijk, C. , P. J. de Jong , and M. L. Peters . 2009. “The Remedial Value of Blushing in the Context of Transgressions and Mishaps.” Emotion (Washington, D.C.) 9: 287–291. 10.1037/a0015081. 19348542

[desc70024-bib-0024] Dong, P. , X. Huang (Irene), and R. S. Wyer . 2013. “The Illusion of Saving Face: How People Symbolically Cope With Embarrassment.” Psychological Science 24, no. 10: 2005–2012. 10.1177/0956797613482946. 23938275

[desc70024-bib-0118] Drummond, P. D. 2013. “Psychophysiology of the Blush.” In The Psychological Significance of the Blush, edited by W. R. Crozier and P. J. de Jong , 15–38. Cambridge University Press.

[desc70024-bib-0026] Drummond, P. D. , G. B. Shapiro , M. Nikolić , and S. M. Bögels . 2020. “Treatment Options for Fear of Blushing.” Current Psychiatry Reports 22, no. 6: 28. 10.1007/s11920-020-01152-5. 32377882

[desc70024-bib-0027] Durán, J. I. , and J.‐M. Fernández‐Dols . 2021. “Do Emotions Result in Their Predicted Facial Expressions? A Meta‐Analysis of Studies on the Co‐Occurrence of Expression and Emotion.” Emotion 21, no. 7: 1550–1569. 10.1037/emo0001015. 34780241

[desc70024-bib-0028] Ekman, P. 1970. “Universal Facial Expressions of Emotion.” California Mental Health Research Digest 8, no. 4: 151–158.

[desc70024-bib-0029] Ekman, P. 1992. “An Argument for Basic Emotions.” Cognition & Emotion 6, no. 3–4: 169–200.

[desc70024-bib-0030] Engelmann, J. M. , E. Herrmann , and M. Tomasello . 2012. “Five‐Year Olds, but Not Chimpanzees, Attempt to Manage Their Reputations.” PLoS ONE 7, no. 10: e48433. 10.1371/journal.pone.0048433. 23119015 PMC3485200

[desc70024-bib-0031] Falk, B. , and R. Dotan . 2008. “Children's Thermoregulation During Exercise in the Heat—A Revisit.” Applied Physiology, Nutrition, and Metabolism 33, no. 2: 420–427. 10.1139/H07-185 18347699

[desc70024-bib-0032] Feinberg, M. , R. Willer , and D. Keltner . 2012. “Flustered and Faithful: Embarrassment as a Signal of Prosociality.” Journal of Personality and Social Psychology 102: 81–97. 10.1037/a0025403. 21928915

[desc70024-bib-0033] Fischer, A. , and U. Hess . 2017. “Mimicking Emotions.” Current Opinion in Psychology 17: 151–155. 10.1016/j.copsyc.2017.07.008. 28950963

[desc70024-bib-0034] Fischer, A. , A. Manstead , I. Lewis , J. Haviland‐Jones , and L. Barrett . 2016. “Social Functions of Emotion and Emotion Regulation.” In Handbook of Emotions (4th edn.), edited by M. Lewis , J. Haviland‐Jones , and L. F. Barrett . Guilford.

[desc70024-bib-0035] Fourie, M. M. , H. G. L. Rauch , B. E. Morgan , G. F. R. Ellis , E. R. Jordaan , and K. G. F. Thomas . 2011. “Guilt and Pride Are Heartfelt, But Not Equally So.” Psychophysiology 48, no. 7: 888–899. 10.1111/j.1469-8986.2010.01157.x. 21143611

[desc70024-bib-0036] Fridlund, A. J. 1991. “Sociality of Solitary Smiling: Potentiation by an Implicit Audience.” Journal of Personality and Social Psychology 60, no. 2: 229–240. 10.1037/0022-3514.60.2.229.

[desc70024-bib-0037] Fridlund, A. J. , K. G. Kenworthy , and A. K. Jaffey . 1992. “Audience Effects in Affective Imagery: Replication and Extension to Dysphoric Imagery.” Journal of Nonverbal Behavior 16, no. 3: 191–212. 10.1007/BF00988034.

[desc70024-bib-0038] Friedman, B. H. , and J. F. Thayer . 2024. “Is Emotion Physiology More Compatible With Discrete, Dimensional, or Appraisal Accounts?” In Emotion Theory: the Routledge Comprehensive Guide. Routledge.

[desc70024-bib-0039] Frijda, N. , and B. Mesquita . 1994. “The Social Roles and Functions of Emotions.” In Emotion and Culture: Empirical Studies of Mutual Influence, edited by S. Kitayama and H. R. Markus , 51–87. American Psychological Association. 10.1037/10152-002.

[desc70024-bib-0040] Gerdemann, S. C. , K. McAuliffe , P. R. Blake , D. B. M. Haun , and R. Hepach . 2022. “The Ontogeny of Children's Social Emotions in Response to (Un)Fairness.” Royal Society Open Science 9, no. 8: 191456. 10.1098/rsos.191456. 36061521 PMC9428536

[desc70024-bib-0041] Gerdemann, S. C. , J. Tippmann , B. Dietrich , J. M. Engelmann , and R. Hepach . 2022. “Young Children Show Negative Emotions After Failing to Help Others.” PLoS ONE 17, no. 4: e0266539. 10.1371/journal.pone.0266539. 35442984 PMC9020688

[desc70024-bib-0042] Gerlach, A. L. , F. H. Wilhelm , K. Gruber , and W. T. Roth . 2001. “Blushing and Physiological Arousability in Social Phobia.” Journal of Abnormal Psychology 110, no. 2: 247–258. 10.1037//0021-843x.110.2.247. 11358019

[desc70024-bib-0043] Gilbert, A. N. , A. J. Fridlund , and J. Sabini . 1987. “Hedonic and Social Determinants of Facial Displays to Odors.” Chemical Senses 12, no. 2: 355–363. 10.1093/chemse/12.2.355.

[desc70024-bib-0044] Goulart, M. , N. C. A. da Costa jr. , E. B. Andrade , and A. A. P. Santos . 2015. “Hedging Against Embarrassment.” Journal of Economic Behavior & Organization 116: 310–318. 10.1016/j.jebo.2015.04.014.

[desc70024-bib-0045] Hamilton, A. F. d. C. , and F. Lind . 2016. “Audience Effects: What Can They Tell Us About Social Neuroscience, Theory of Mind and Autism?” Culture and Brain 4, no. 2: 159–177. 10.1007/s40167-016-0044-5. 27867833 PMC5095155

[desc70024-bib-0046] Harris, C. R. 2001. “Cardiovascular Responses of Embarrassment and Effects of Emotional Suppression in a Social Setting.” Journal of Personality and Social Psychology 81, no. 5: 886–897. 10.1037/0022-3514.81.5.886. 11708564

[desc70024-bib-0047] Harris, P. L. 1983. “Children's Understanding of the Link Between Situation and Emotion.” Journal of Experimental Child Psychology 36, no. 3: 490–509. 10.1016/0022-0965(83)90048-6.

[desc70024-bib-0048] Haun, D. B. M. , Y. Rekers , and M. Tomasello . 2014. “Children Conform to the Behavior of Peers; Other Great Apes Stick With What They Know.” Psychological Science 25, no. 12: 2160–2167. 10.1177/0956797614553235. 25355648

[desc70024-bib-0049] Haun, D. B. M. , and M. Tomasello . 2011. “Conformity to Peer Pressure in Preschool Children.” Child Development 82, no. 6: 1759–1767. 10.1111/j.1467-8624.2011.01666.x. 22023172

[desc70024-bib-0050] Holodynski, M. 2004. “The Miniaturization of Expression in the Development of Emotional Self‐Regulation.” Developmental Psychology 40, no. 1: 16–28. 10.1037/0012-1649.40.1.16. 14700461

[desc70024-bib-0051] Holodynski, M. 2006. “Die Entwicklung der Leistungs‐motivation im Vorschulalter: Soziale Bewertungen und Ihre Auswirkung auf Stolz‐, Scham‐ und Ausdauerreaktionen. [The Development of Achievement Motivation in Preschool‐age Children. Social Evaluations and Their Effect on Pride, Shame, and Perseverance Reactions.].” Zeitschrift Für Entwicklungspsychologie Und Pädagogische Psychologie 38, no. 1: 2–17. 10.1026/0049-8637.38.1.2.

[desc70024-bib-0115] Jakobs, E. , A. S. R. Manstead , and A. H. Fischer . 1999. “Social Motives and Emotional Feelings as Determinants of Facial Displays: The Case of Smiling.” Personality and Social Psychology Bulletin 25, no. 4: 424–435. 10.1177/0146167299025004003.

[desc70024-bib-0111] JASP Team . 2024. JASP (Version 0.19. 3)[Computer software].

[desc70024-bib-0052] Kampis, D. , and V. Southgate . 2020. “Altercentric Cognition: How Others Influence Our Cognitive Processing.” Trends in Cognitive Sciences 24, no. 11: 945–959. 10.1016/j.tics.2020.09.003. 32981846

[desc70024-bib-0053] Keltner, D. , and B. N. Buswell . 1996. “Evidence for the Distinctness of Embarrassment, Shame, and Guilt: A Study of Recalled Antecedents and Facial Expressions of Emotion.” Cognition and Emotion 10, no. 2: 155–171. 10.1080/026999396380312.

[desc70024-bib-0054] Keltner, D. , and B. N. Buswell . 1997. “Embarrassment: Its Distinct Form and Appeasement Functions.” Psychological Bulletin 122, no. 3: 250–270. 10.1037/0033-2909.122.3.250. 9354148

[desc70024-bib-0055] Kraut, R. E. , and R. E. Johnston . 1979. “Social and Emotional Messages of Smiling: An Ethological Approach.” Journal of Personality and Social Psychology 37, no. 9: 1539–1553. 10.1037/0022-3514.37.9.1539.

[desc70024-bib-0056] Kreibig, S. D. 2010. “Autonomic Nervous System Activity in Emotion: A Review.” Biological Psychology 84, no. 3: 394–421. 10.1016/j.biopsycho.2010.03.010. 20371374

[desc70024-bib-0057] Kret, M. E. 2015. “Emotional Expressions Beyond Facial Muscle Actions. A Call for Studying Autonomic Signals and Their Impact on Social Perception.” Frontiers in Psychology 6: 711. 10.3389/fpsyg.2015.00711. 26074855 PMC4443639

[desc70024-bib-0058] Kromm, H. , M. Färber , and M. Holodynski . 2015. “Felt or False Smiles? Volitional Regulation of Emotional Expression in 4‐, 6‐, and 8‐Year‐Old Children.” Child Development 86, no. 2: 579–597. 10.1111/cdev.12315. 25382704

[desc70024-bib-0059] Leary, M. R. 2004. “Digging Deeper: The Fundamental Nature of “Self‐conscious” Emotions.” Psychological Inquiry 15, no. 2: 129–131.

[desc70024-bib-0060] Leary, M. R. 2019. Self‐Presentation: Impression Management and Interpersonal Behavior. Routledge. 10.4324/9780429497384.

[desc70024-bib-0061] Leary, M. R. , T. W. Britt , W. D. Cutlip , and J. L. Templeton . 1992. “Social Blushing.” Psychological Bulletin 112, no. 3: 446. 1438638 10.1037/0033-2909.112.3.446

[desc70024-bib-0062] Leary, M. R. , and S. Meadows . 1991. “Predictors, Elicitors, and Concomitants of Social Blushing.” Journal of Personality and Social Psychology 60, no. 2: 254–262. 10.1037/0022-3514.60.2.254.

[desc70024-bib-0063] Lewis, M. 2007. “Self‐Conscious Emotional Development.” In The Self‐Conscious Emotions: Theory and Research, edited by J. L. Tracy , R. W. Robins , and J. P. Tangney , 134–149. The Guilford Press.

[desc70024-bib-0064] Lewis, M. 2008. “Self‐Conscious Emotions: Embarrassment, Pride, Shame, and Guilt.” In Handbook of Emotions, 742–756. 3rd ed. The Guilford Press.

[desc70024-bib-0065] Lewis, M. , and D. Ramsay . 2002. “Cortisol Response to Embarrassment and Shame.” Child Development 73, no. 4: 1034–1045. 10.1111/1467-8624.00455. 12146731

[desc70024-bib-0066] Lewis, M. , C. Stanger , M. W. Sullivan , and P. Barone . 1991. “Changes in Embarrassment as a Function of Age, Sex and Situation.” British Journal of Developmental Psychology 9, no. 4: 485–492. 10.1111/j.2044-835X.1991.tb00891.x.

[desc70024-bib-0067] Lewis, M. , M. W. Sullivan , C. Stanger , and M. Weiss . 1989. “Self Development and Self‐conscious Emotions.” Child Development 146–156. 2702864

[desc70024-bib-0069] Lewis, M. , and M. Wolan Sullivan . 2005. “The Development of Self‐Conscious Emotions.” In Handbook of Competence and Motivation, 185–201. Guilford Publications.

[desc70024-bib-0070] Mahmood, T. , and M. Xie . 2019. “Models and Monitoring of Zero‐Inflated Processes: The Past and Current Trends.” Quality and Reliability Engineering International 35, no. 8: 2540–2557. 10.1002/qre.2547.

[desc70024-bib-0071] Malatesta, C. Z. , and J. M. Haviland . 1982. “Learning Display Rules: The Socialization of Emotion Expression in Infancy.” Child Development 53, no. 4: 991–1003. 7128264

[desc70024-bib-0114] Manokara, K. , A. Fischer , and D. Sauter . 2023. “Display Rules Differ Between Positive Emotions: Not All That Feels Good Looks Good.” Emotion 23, no. 1: 243–260. 10.1037/emo0001078. 35266776

[desc70024-bib-0117] Matsumoto, D. , Seung Hee Yoo , and J. Fontaine . 2008. “Mapping Expressive Differences Around the World: The Relationship Between Emotional Display Rules and Individualism Versus Collectivism.” Journal of Cross‐Cultural Psychology 39, no. 1: 55–74. 10.1177/0022022107311854.

[desc70024-bib-0072] Mauss, I. , R. Levenson , L. McCarter , F. Wilhelm , and J. Gross . 2005. “The Tie That Binds? Coherence Among Emotion Experience, Behavior, and Physiology.” Emotion (Washington, D.C.) 5, no. 2: 175. 10.1037/1528-3542.5.2.175. 15982083

[desc70024-bib-0073] Mesquita, B. , and B. Parkinson . 2024. “Social Constructionist Theories of Emotions.” In Emotion Theory: The Routledge Comprehensive Guide. Routledge.

[desc70024-bib-0074] Miller, R. S. 1995. “Embarrassment and Social Behavior.” In Self‐Conscious Emotions: The Psychology of Shame, Guilt, Embarrassment, and Pride, 322–339. Guilford Press.

[desc70024-bib-0075] Mulkens, S. , P. J. De Jong , and S. M. Bögels . 1997. “High Blushing Propensity: Fearful Preoccupation or Facial Coloration?” Personality and Individual Differences 22, no. 6: 817–824. 10.1016/S0191-8869(97)00008-1.

[desc70024-bib-0076] Mulkey, S. B. , and A. J. du Plessis . 2019. “Autonomic Nervous System Development and Its Impact on Neuropsychiatric Outcome.” Pediatric Research 85, no. 2: 120–126. 10.1038/s41390-018-0155-0. 30166644 PMC6353676

[desc70024-bib-0077] Nikolić, M. , E. Brummelman , C. Colonnesi , W. de Vente , and S. M. Bögels . 2018. “When Gushing Leads to Blushing: Inflated Praise Leads Socially Anxious Children to Blush.” Behaviour Research and Therapy 106: 1–7. 10.1016/j.brat.2018.04.003. 29705596

[desc70024-bib-0078] Nikolić, M. , W. De Vente , C. Colonnesi , and S. M. Bögels . 2016. “Autonomic Arousal in Children of Parents With and Without Social Anxiety Disorder: A High‐Risk Study.” Journal of Child Psychology and Psychiatry 57, no. 9: 1047–1055. 10.1111/jcpp.12563. 27133173

[desc70024-bib-0079] Nikolić, M. , L. J. Hannigan , G. Krebs , A. Sterne , A. M. Gregory , and T. C. Eley . 2022. “Aetiology of Shame and Its Association With Adolescent Depression and Anxiety: Results From a Prospective Twin and Sibling Study.” Journal of Child Psychology and Psychiatry 63, no. 1: 99–108. 10.1111/jcpp.13465. 34132398 PMC9292396

[desc70024-bib-0080] Nikolić, M. , L. van der Storm , C. Colonnesi , E. Brummelman , K. J. Kan , and S. Bögels . 2019. “Are Socially Anxious Children Poor or Advanced Mindreaders?” Child Development 90, no. 4: 1424–1441. 10.1111/cdev.13248. 31099053 PMC6852401

[desc70024-bib-0082] Peirce, J. , J. R. Gray , S. Simpson , et al. 2019. “PsychoPy2: Experiments in Behavior Made Easy.” Behavior Research Methods 51, no. 1: 195–203. 10.3758/s13428-018-01193-y. 30734206 PMC6420413

[desc70024-bib-0083] Penttilä, J. , A. Helminen , T. Jartti , et al. 2001. “Time Domain, Geometrical and Frequency Domain Analysis of Cardiac Vagal Outflow: Effects of Various Respiratory Patterns.” Clinical Physiology (Oxford, England) 21, no. 3: 365–376. 10.1046/j.1365-2281.2001.00337.x. 11380537

[desc70024-bib-0084] Porges, S. W. 2009. “The Polyvagal Theory: New Insights Into Adaptive Reactions of the Autonomic Nervous System.” Cleveland Clinic Journal of Medicine 76, no. Suppl 2: S86–S90. 10.3949/ccjm.76.s2.17. 19376991 PMC3108032

[desc70024-bib-0086] Recio, G. , and W. Sommer . 2018. “Copycat of Dynamic Facial Expressions: Superior Volitional Motor Control for Expressions of Disgust.” Neuropsychologia 119: 512–523. 10.1016/j.neuropsychologia.2018.08.027. 30176302

[desc70024-bib-0087] Reddy, V. 2004. “Feeling Shy and Showing‐Off: Self‐Conscious Emotions Must Regulate Self‐Awareness.” In Emotional Development: Recent Research Advances, edited by J. Nadel and D. Muir , 0. Oxford University Press. 10.1093/acprof:oso/9780198528845.003.0007.

[desc70024-bib-0088] Russell, J. A. , and L. F. Barrett . 1999. “Core Affect, Prototypical Emotional Episodes, and Other Things Called Emotion: Dissecting the Elephant.” Journal of Personality and Social Psychology 76, no. 5: 805–819. 10.1037/0022-3514.76.5.805. 10353204

[desc70024-bib-0089] Satow, K. L. 1975. “Social Approval and Helping.” Journal of Experimental Social Psychology 11, no. 6: 501–509. 10.1016/0022-1031(75)90001-3.

[desc70024-bib-0090] Schachter, S. , and J. Singer . 1962. “Cognitive, Social, and Physiological Determinants of Emotional State.” Psychological Review 69, no. 5: 379–399. 10.1037/h0046234. 14497895

[desc70024-bib-0091] Schandry, R. , and E. Poth . 1983. “Eine Experimentelle Untersuchung zur Psychologie und Physiologie des Errötens. [An Experimental Investigation on the Psychology and Physiology of Blushing.].” Psychologische Beitrage 25: 503–514.

[desc70024-bib-0092] Shearn, D. , E. Bergman , K. Hill , A. Abel , and L. Hinds . 1990. “Facial Coloration and Temperature Responses in Blushing.” Psychophysiology 27, no. 6: 687–693. 10.1111/j.1469-8986.1990.tb03194.x. 2100354

[desc70024-bib-0093] Shearn, D. , E. Bergman , K. Hill , A. Abel , and L. Hinds . 1992. “Blushing as a Function of Audience Size.” Psychophysiology 29, no. 4: 431–436. 10.1111/j.1469-8986.1992.tb01716.x. 1410174

[desc70024-bib-0094] Silvetti, M. S. , F. Drago , and P. Ragonese . 2001. “Heart Rate Variability in Healthy Children and Adolescents Is Partially Related to Age and Gender.” International Journal of Cardiology 81, no. 2: 169–174. 10.1016/S0167-5273(01)00537-X. 11744133

[desc70024-bib-0095] Sjak‐Shie, E. E. 2022. PhysioData Toolbox (Version 0.6.3) [Computer Software]. https://PhysioDataToolbox.leidenuniv.nl.

[desc70024-bib-0113] Somerville, L. H. , R. M. Jones , E. J. Ruberry , J. P. Dyke , G. Glover , and B. J. Casey . 2013. “The Medial Prefrontal Cortex and the Emergence of Self‐Conscious Emotion in Adolescence.” Psychological Science 24, no. 8: 1554–1562. 10.1177/0956797613475633. 23804962 PMC3742683

[desc70024-bib-0097] Stipek, D. 1995. “The Development of Pride and Shame in Toddlers.” In Self‐Conscious Emotions: The Psychology of Shame, Guilt, Embarrassment, and Pride, 237–252. Guilford Press.

[desc70024-bib-0098] Stipek, D. , S. Recchia , S. McClintic , and M. Lewis . 1992. “Self‐Evaluation in Young Children.” Monographs of the Society for Research in Child Development 57, no. 1: 1–95. 10.2307/1166190. 1560797

[desc70024-bib-0099] Stoeber, J. , R. A. Harris , and P. S. Moon . 2007. “Perfectionism and the Experience of Pride, Shame, and Guilt: Comparing Healthy Perfectionists, Unhealthy Perfectionists, and Non‐Perfectionists.” Personality and Individual Differences 43, no. 1: 131–141. 10.1016/j.paid.2006.11.012.

[desc70024-bib-0100] Sznycer, D. 2019. “Forms and Functions of the Self‐Conscious Emotions.” Trends in Cognitive Sciences 23, no. 2: 143–157. 10.1016/j.tics.2018.11.007. 30583948

[desc70024-bib-0102] Tracy, J. L. , and R. W. Robins . 2004. “Keeping the Self in Self‐Conscious Emotions: Further Arguments for a Theoretical Model.” Psychological Inquiry 15, no. 2: 171–177.

[desc70024-bib-0103] Tracy, J. L. , and R. W. Robins . 2007. “Emerging Insights Into the Nature and Function of Pride.” Current Directions in Psychological Science 16, no. 3: 147–150. 10.1111/j.1467-8721.2007.00493.x.

[desc70024-bib-0104] Tracy, J. L. , and A. C. Weidman . 2021. “The Self‑Conscious and Social Emotions: A Personality and Social Functionalist Account.” In Handbook of Personality: Theory and Research, 504–522. 4th ed. The Guilford Press.

[desc70024-bib-0105] Triplett, N. 1898. “The Dynamogenic Factors in Pacemaking and Competition.” American Journal of Psychology 9, no. 4: 507–533. 10.2307/1412188.

[desc70024-bib-0106] van Kleef, G. A. , A. Cheshin , A. H. Fischer , and I. K. Schneider . 2016. “Editorial: The Social Nature of Emotions.” Frontiers in Psychology 7: 896. https://www.frontiersin.org/articles/10.3389/fpsyg.2016.00896. 27378990 10.3389/fpsyg.2016.00896 PMC4906017

[desc70024-bib-0107] van Osch, Y. , M. Zeelenberg , S. M. Breugelmans , and M. J. Brandt . 2019. “Show or Hide Pride? Selective Inhibition of Pride Expressions as a Function of Relevance of Achievement Domain.” Emotion (Washington, D.C.) 19, no. 2: 334–347. 10.1037/emo0000437. 29878803

[desc70024-bib-0108] Webster, J. M. , J. Duvall , L. M. Gaines , and R. H. Smith . 2003. “The Roles of Praise and Social Comparison Information in the Experience of Pride.” Journal of Social Psychology 143, no. 2: 209–232. 12735519 10.1080/00224540309598441

[desc70024-bib-0109] Westenberg, P. M. , M. J. Drewes , A. W. Goedhart , B. M. Siebelink , and P. D. A. Treffers . 2004. “A Developmental Analysis of Self‐Reported Fears in Late Childhood Through Mid‐Adolescence: Social‐Evaluative Fears on the Rise?” Journal of Child Psychology and Psychiatry, and Allied Disciplines 45, no. 3: 481–495. 10.1111/j.1469-7610.2004.00239.x. 15055368

[desc70024-bib-0110] Williams, L. A. , and D. DeSteno . 2008. “Pride and Perseverance: The Motivational Role of Pride.” Journal of Personality and Social Psychology 94: 1007–1017. 10.1037/0022-3514.94.6.1007. 18505314

[desc70024-bib-0116] Zeman, J. and J. Garber . 1996. “Display Rules for Anger, Sadness, and Pain: It Depends on who is Watching.” Child Development 67, no. 3: 957–973. 8706538

