# Supplementary material for: Putting the Social in Emotions: The Effect of Audience Presence on Pride and Embarrassment Across Ontogeny
Source: Dev Sci. 2025 May 19;28(4):e70024. doi: 10.1111/desc.70024 (PMC12087427; doi:10.1111/desc.70024)
Supplement: Supplementary file 1 — Supporting information [file DESC-28-e70024-s004.docx]

**Pre-Registered Data Analyses**

To examine the effects of age group, audience condition and emotion induction task on our physiological response (SCL, HRV and cheek temperature) and embarrassment/pride nonverbal emotional expression variables, we used a series of (generalized) linear mixed models. Here, we entered emotion induction task (pride versus embarrassment), audience presence condition (alone versus audience) and age group (adults, older children, younger children), as well as their interactions, into the model as fixed factors using dummy coding. We set the adult group, the pride task and the audience condition as the reference categories to which the rest of the levels of the model were compared. The random effect structure was emotion induction task (level 1) nested within individuals (level 2). We included random intercepts for participants, as we expected between-participant variation in physiological response and nonverbal expressions, but did not include any random slopes. We inspected all variables for outliers before running our models, and, where possible, winsorized values greater or less than three standard deviations from the mean, preserving rank order.

For the physiological data, we fitted the models using a Gaussian distribution and identity link, given that data were approximately normally distributed. For the pride and embarrassment nonverbal expression data, we used zero-inflated Gamma distributions with a log link, as preliminary analyses revealed that nonverbal expression duration scores were highly positively skewed (many participants displayed very low levels of embarrassment/pride, with a high proportion of zeros) (Mahmood & Xie, 2019). This was particularly marked for pride facial expressions. As such, in lieu of transforming our data, which had minimal impact on the distribution of residuals, we opted to use zero-inflated gamma GLMMs which matched the distribution of our dependent variable (namely, non-negative and positively skewed) (Brooks et al., 2017).

As is common, for all models, we dropped non-significant interaction terms one-by-one, starting with higher order interaction terms (Prochazkova et al., 2018, 2022). In this case, we examined log-likelihoods to determine if dropping said interaction term improved model fit, or significantly worsened it, in which case the non-significant factor was kept. All analyses were conducted using R Studio (R Studio Team, 2023). We fit models using the glmmTMB package (Brooks et al., 2017). To examine the significant of main- and interaction-effects, we used Type-III Wald tests using the car package (Fox et al., 2019). Finally, in the case of significant model terms, Bonferroni-corrected post-hoc pairwise comparisons between factor levels (and their interactions) were derived by comparing estimated marginal means using the emmeans package (Lenth et al., 2019).

Finally, due to a programming error, *n* = 11 older children and *n* = 1 adult heard two rounds of false positive feedback during the pride induction. In lieu of excluding these participants’ data in the pride task, we ran the analyses with these participants included and excluded to see whether they exerted undue influence on the findings in any way. The interpretation of the findings was identical in both cases, suggesting that this was not the case. As such, we report the results of the analyses including all participants below.

***Did audience presence influence emotional responding across the embarrassment and pride tasks?***

When considering our pre-registered models, across all physiological and nonverbal emotional expression measures, we found no effect of audience presence on participants’ emotional responding (Tables S1-S5). This result was consistent across all age groups and emotion induction tasks – suggesting that audience presence did not influence participants’ responding when considering both induction tasks together.

***Were there age-related differences in emotional responding across the viewing tasks?***

We found a significant interaction effect between age group and emotion induction task on participants’ cheek temperature change scores, *χ^2^*(2) = 26.72, *p* < .001 (Table S1). Pairwise contrasts are shown in Table S2 and revealed that this interaction effect was such that both younger and older children demonstrated a significantly greater cheek temperature increase in the pride task compared to adults, *t*(397) = -4.38, *p* < .001, *t*(397) = -2.50, *p* = .039, respectively. In the embarrassment task, no differences between children and adults could be established, *p*’s > .05. Similarly, in terms of skin conductance level change scores, we found an interaction between age and emotion induction task, *χ^2^*(2) = 15.95, *p* < .001 (Table S8). This effect was such that the pride task, but not the embarrassment task, resulted in a significantly greater decrease in skin conductance level in adults compared to young children, *t*(411) = -4.54 *p* < .001 (Table S4). No other significant differences across age groups and emotion induction tasks could be established, all *p’*s > .05.

The model on heart rate variability change scores revealed a significant main effect of age group, *χ^2^*(2) = 16.94, *p* < .001 (Table S5), with adults demonstrating significantly lower heart rate variability change scores compared to younger children across tasks, *t*(409) = -4.08, *p* < .001 (Table S6). Older children also displayed lower heart rate variability change scores compared to younger children across tasks, *t*(409) = -2.63, *p* = .027. In terms of nonverbal expressions of embarrassment, the model revealed a significant interaction effect between age and emotion induction task, *χ^2^*(2) = 24.35, *p <* .001 (Table S7). Estimated mean contrasts (Table S8) revealed that older children displayed significantly more nonverbal embarrassment expressions than younger children while watching the embarrassing video, *t*(409) = 3.92, *p* < .001 The duration of nonverbal embarrassment expressions was similar in the embarrassment task across all other age group combinations, all *p*’s > .05. When considering nonverbal embarrassment displays in the pride task, both younger and older children appeared to show more embarrassment compared to adults, *t*(409) = 3.40, *p* = .002, *t*(409) = 2.97, *p* = .010, respectively. Finally, the model on nonverbal expressions of pride revealed no age-related differences in nonverbal pride expressions, *χ^2^*(2) = 1.18, *p* = .553 (Table S9). Participants’ emotional responding across all measures and across age is plotted in Figure 1.

**Figure 1**

*Bar Plots Indicating Participants’ Emotional Responding Across Age and Across Both Pride and Embarrassment Induction Tasks*

**
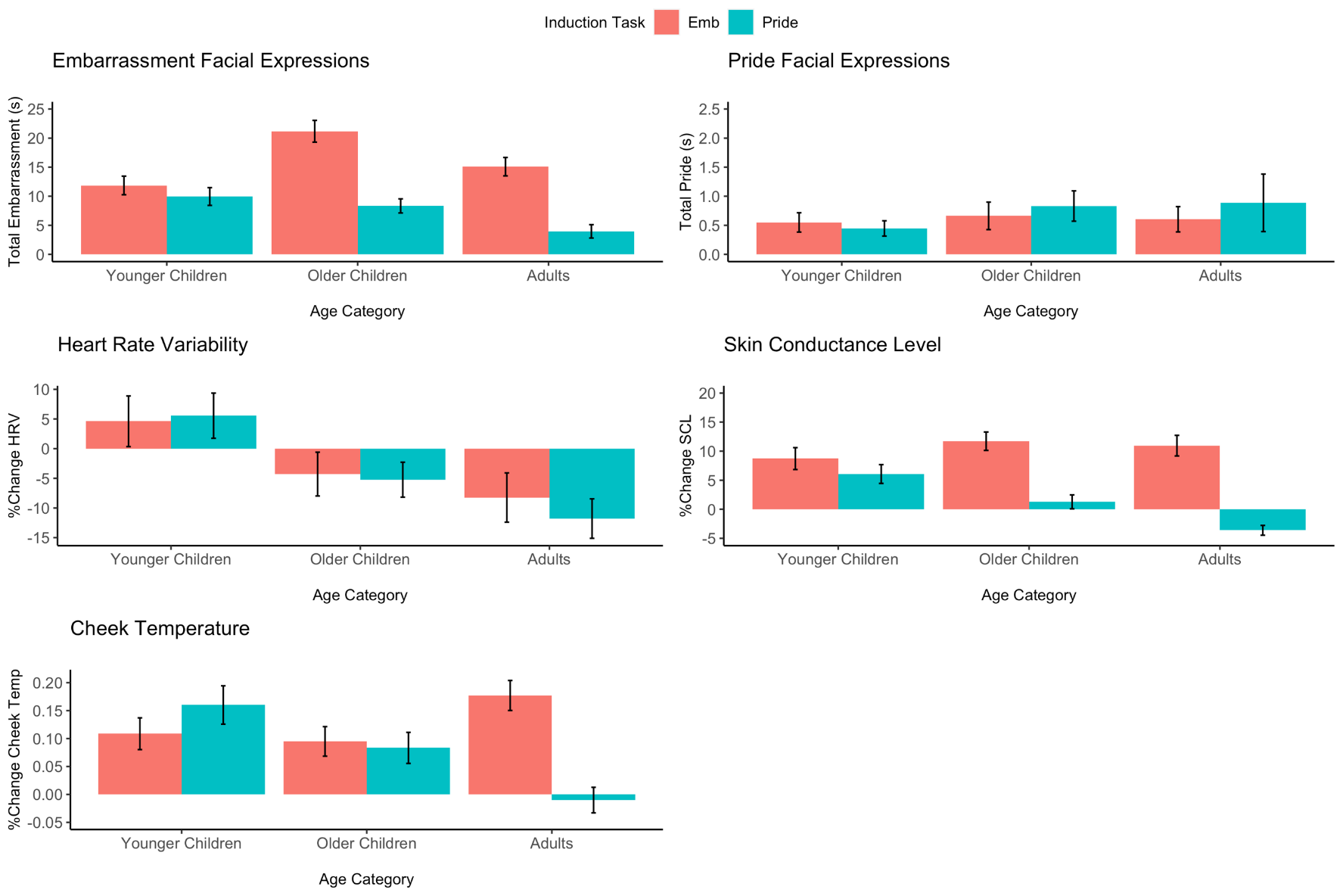
**

*Note.* Error bars represent standard error

**Table S1.**

Results of Linear Mixed Model Predicting Cheek Temperature Change Scores by Age Category, Emotion Induction Task and Audience Condition

|  | |  | **Skin Temperature Change** | | | | | |
| --- | --- | --- | --- | --- | --- | --- | --- | --- |
| *Predictors* | | *Estimates* | | *df1* | *df2* | *χ2* | *z* | *p* |
| (Intercept) | | 0.01 | | 1 | 397 | 0.14 | 0.37 | .710 |
| Age Category | |  | | 2 | 397 | 19.42 |  | **<.001** |
| *Adults (Reference)* | |  | |  |  |  |  |  |
| *Older Children* | | 0.09 | |  |  |  | 2.50 | **.012** |
| *Younger Children* | | 0.17 | |  |  |  | 4.38 | **<.001** |
| Induction Task | |  | | 1 | 397 | 32.73 |  | **<.001** |
| *Pride (Reference)* | |  | |  |  |  |  |  |
| *Embarrassment* | | 0.19 | |  |  |  | 5.72 | **<.001** |
| Audience Condition | |  | | 1 | 397 | 2.98 |  | .084 |
| *Audience (Reference)* | |  | |  |  |  |  |  |
| *Alone* | | -0.04 | |  |  |  | -1.73 | .084 |
| Induction * Age Category | |  | | 2 | 397 | 26.72 |  | **<.001** |
| *Older Children * Embarrassment* | | -0.18 | |  |  |  | -3.76 | **<.001** |
| *Younger Children * Embarrassment* | | -0.24 | |  |  |  | -4.90 | **<.001** |
|  | **Random Effects** | | | | | | | |
| σ^2^ | |  | 0.04 | | | | | |
| τ_00_ _pp_ | |  | 0.01 | | | | | |
| ICC | |  | 0.22 | | | | | |
| N _pp_ | |  | 203 | | | | | |
| Observations | |  | 406 | | | | | |
| Marginal R^2^ / Conditional R^2^ | |  | 0.079/ 0.285 | | | | | |

**Table S2.**

Results of Estimated Mean Contrasts Between Age Group and Emotion Induction Task Factor Levels in the Linear Mixed Model Predicting Cheek Temperature Change Scores.

| Between age group | *Estimate* | *t* | *p* | *Estimate* | *t* | *p* |
| --- | --- | --- | --- | --- | --- | --- |
|  | Pride | | | Embarrassment | | |
| Adult – Older Child | -0.09 | -2.50 | **.039** | 0.08 | 2.18 | .089 |
| Adult – Younger Child | -0.17 | -4.38 | **<.001** | 0.07 | 1.72 | .257 |
| Older Child – Younger Child | -0.08 | -1.96 | .152 | -0.01 | -0.37 | 1.00 |
| Within age group | Pride – Embarrassment | | |  |  |  |
| Adult | -0.19 | -5.72 | **<.001** |  |  |  |
| Older Child | -0.01 | -0.34 | .732 |  |  |  |
| Younger Child | -0.05 | 1.43 | .155 |  |  |  |

*Note.* P-values Bonferroni corrected

**Table S3.**

Results of Linear Mixed Model Predicting Skin Conductance Level Change Scores by Age Category, Emotion Induction Task and Audience Condition

|  | |  | **Skin Conductance Change** | | | | | |
| --- | --- | --- | --- | --- | --- | --- | --- | --- |
| *Predictors* | | *Estimates* | | *df1* | *df2* | *χ2* | *z* | *p* |
| (Intercept) | | -3.66 | | 1 | 411 | 5.26 | -2.29 | **.022** |
| Age Category | |  | | 2 | 411 | 20.63 |  | **<.001** |
| *Adults (Reference)* | |  | |  |  |  |  |  |
| *Older Children* | | 4.88 | |  |  |  | 2.31 | **.021** |
| *Younger Children* | | 9.70 | |  |  |  | 4.54 | **<.001** |
| Induction Task | |  | | 1 | 411 | 48.66 |  | **<.001** |
| *Pride (Reference)* | |  | |  |  |  |  |  |
| *Embarrassment* | | 14.57 | |  |  |  | 6.98 | **<.001** |
| Audience Condition | |  | | 1 | 411 | 0.00 |  | .944 |
| *Audience (Reference)* | |  | |  |  |  |  |  |
| *Alone* | | 0.09 | |  |  |  | 0.07 | .944 |
| Induction * Age Category | |  | | 2 |  | 15.95 |  | **<.001** |
| *Older Children * Embarrassment* | | -4.12 | |  |  |  | -1.38 | .168 |
| *Younger Children * Embarrassment* | | -11.92 | |  |  |  | -3.95 | **<.001** |
|  | **Random Effects** | | | | | | | |
| σ^2^ | |  | 159.31 | | | | | |
| τ_00_ _pp_ | |  | 0.00 | | | | | |
| ICC | |  | 0.00 | | | | | |
| N _pp_ | |  | 210 | | | | | |
| Observations | |  | 420 | | | | | |
| Marginal R^2^ / Conditional R^2^ | |  | 0.162 / NA | | | | | |

**Table S4.**

Results of Estimated Mean Contrasts Between Age Group and Emotion Induction Task Factor Levels in the Linear Mixed Model Predicting Skin Conductance Level Change Scores.

| Between age group | *Estimate* | *t* | *p* | *Estimate* | *t* | *p* |
| --- | --- | --- | --- | --- | --- | --- |
|  | Pride | | | Embarrassment | | |
| Adult – Older Child | -4.88 | -2.31 | .064 | -0.77 | -0.36 | 1.00 |
| Adult – Younger Child | -9.70 | -4.54 | **<.001** | 2.23 | 1.04 | .893 |
| Older Child – Younger Child | -4.81 | -2.23 | .079 | 2.99 | 1.39 | .499 |
| Within age group | Pride – Embarrassment | | |  |  |  |
| Adult | -14.57 | -6.98 | **<.001** |  |  |  |
| Older Child | -10.45 | -4.90 | **<.001** |  |  |  |
| Younger Child | -2.65 | -1.22 | .225 |  |  |  |

*Note.* P-values Bonferonni corrected

**Table S5.**

Results of Linear Mixed Model Predicting Heart Rate Variability Change Scores by Age Category, Emotion Induction Task and Audience Condition

|  | |  | **Heart Rate Variability Change** | | | | | |
| --- | --- | --- | --- | --- | --- | --- | --- | --- |
| *Predictors* | | *Estimates* | | *df1* | *df2* | *χ2* | *z* | *p* |
| (Intercept) | | -10.89 | | 1 | 409 | 10.82 | -3.29 | **.001** |
| Age Category | |  | | 2 | 409 | 16.94 |  | **<.001** |
| *Adults (Reference)* | |  | |  |  |  |  |  |
| *Older Children* | | 5.25 | |  |  |  | 1.44 | .149 |
| *Younger Children* | | 15.11 | |  |  |  | 4.08 | **<.001** |
| Induction Task | |  | | 1 | 409 | 0.18 |  | .674 |
| *Pride (Reference)* | |  | |  |  |  |  |  |
| *Embarrassment* | | 1.27 | |  |  |  | 0.42 | .674 |
| Audience Condition | |  | | 1 | 409 | 0.03 |  | .870 |
| *Audience (Reference)* | |  | |  |  |  |  |  |
| *Alone* | | 0.49 | |  |  |  | 0.16 | .870 |
|  | **Random Effects** | | | | | | | |
| σ^2^ | |  | 945.48 | | | | | |
| τ_00_ _pp_ | |  | 0.00 | | | | | |
| N _pp_ | |  | 208 | | | | | |
| Observations | |  | 416 | | | | | |
| Marginal R^2^ / Conditional R^2^ | |  | 0.04 / NA | | | | | |

**Table S6**

Results of Estimated Mean Contrasts Between Age Group in the Linear Mixed Model Predicting Heart Rate Variability Change Scores.

| Between age group | *Estimate* | *t* | *p* |
| --- | --- | --- | --- |
|  |  | | |
| Adult – Older Child | -5.25 | -1.44 | .448 |
| Adult – Younger Child | -15.11 | -4.08 | **<.001** |
| Older Child – Younger Child | -9.86 | -2.63 | **.027** |

*Note.* P-values Bonferonni corrected

**Table S7.**

Results of Zero-Inflated Gamma Generalized Linear Mixed Model Predicting Embarrassment Nonverbal Expression Duration by Age Group, Emotion Induction Task and Audience Condition

|  | |  | **Embarrassment Duration** | | | | | |
| --- | --- | --- | --- | --- | --- | --- | --- | --- |
| *Predictors* | | *Estimates* | | *df1* | *df2* | *χ2* | *z* | *p* |
| (Intercept) | | 4.03 | | 1 | 410 | 64.18 | 8.01 | **<.001** |
| Age Category | |  | | 2 | 410 | 13.20 |  | **.001** |
| *Adults (Reference)* | |  | |  |  |  |  |  |
| *Older Children* | | 1.88 | |  |  |  | 2.97 | **.003** |
| *Younger Children* | | 2.05 | |  |  |  | 3.40 | **.001** |
| Induction Task | |  | | 1 | 410 | 51.07 |  | **<.001** |
| *Pride (Reference)* | |  | |  |  |  |  |  |
| *Embarrassment* | | 3.53 | |  |  |  | 7.15 | **<.001** |
| Audience Condition | |  | | 1 | 410 | 0.02 |  | .878 |
| *Audience (Reference)* | |  | |  |  |  |  |  |
| *Alone* | | 1.02 | |  |  |  | 0.15 | .878 |
| Induction*Age Category | |  | | 2 | 410 | 24.35 |  | **<.001** |
| *Older Children * Embarrassment* | | 0.78 | |  |  |  | -1.05 | .294 |
| *Younger Children * Embarrassment* | | 0.34 | |  |  |  | -4.59 | **<.001** |
| **Zero-Inflated Model** | |  | |  |  |  |  |  |
| (Intercept) | | 0.17 | |  |  |  | -12.78 | **<.001** |
|  | **Random Effects** | | | | | | | |
| σ^2^ | |  | 0.64 | | | | | |
| τ_00_ _pp_ | |  | 0.43 | | | | | |
| ICC | |  | 0.40 | | | | | |
| N _pp_ | |  | 213 | | | | | |
| Observations | |  | 419 | | | | | |
| Marginal R^2^ / Conditional R^2^ | |  | 0.203/0.522 | | | | | |

**Table S8.**

Results of Estimated Mean Contrasts Between Age Group and Induction Task Factor Levels in the Zero-Inflated Gamma Generalized Linear Mixed Model Predicting Embarrassment Nonverbal Expression Duration

| Between age group | *Ratio* | *t-ratio* | *p* | *Ratio* | *t-ratio* | *p* |
| --- | --- | --- | --- | --- | --- | --- |
|  | Pride Task | | | Embarrassment Task | | |
| Adult – Older Child | 0.53 | -2.97 | **.010** | 0.68 | -2.09 | .111 |
| Adult – Younger Child | 0.49 | -3.40 | **.002** | 1.44 | 1.92 | .167 |
| Older Child – Younger Child | 0.92 | -0.44 | 1.00 | 2.12 | 3.92 | **<.001** |
| Within age group | Pride – Embarrassment | | |  |  |  |
| Adult | 0.28 | -7.15 | **<.001** |  |  |  |
| Older Child | 0.37 | -6.52 | **<.001** |  |  |  |
| Younger Child | 0.84 | -1.13 | .260 |  |  |  |

*Note.* P-values Bonferonni corrected. Tests are performed on the log scale.

**Table S9.**

Results of Zero-Inflated Gamma Generalized Linear Mixed Model Predicting Pride Nonverbal Expression Duration by Age Group, Emotion Induction Task and Audience Condition

|  |  | **Pride Duration** | | | | | |
| --- | --- | --- | --- | --- | --- | --- | --- |
| *Predictors* | *Estimates* | | *df1* | *df2* | *χ2* | *z* | *p* |
| (Intercept) | 2.96 | | 1 | 424 | 8.90 | 2.98 | **.003** |
| Age Category |  | | 2 | 424 | 1.18 |  | .553 |
| *Adults (Reference)* |  | |  |  |  |  |  |
| *Older Children* | 0.88 | |  |  |  | -0.40 | .689 |
| *Younger Children* | 0.70 | |  |  |  | -1.04 | .300 |
| Induction Task |  | | 1 | 424 | 0.12 |  | .729 |
| *Pride (Reference)* |  | |  |  |  |  |  |
| *Embarrassment* | 0.92 | |  |  |  | -0.35 | .729 |
| Audience Condition |  | | 1 | 424 | 0.20 |  | .657 |
| *Audience (Reference)* |  | |  |  |  |  |  |
| *Alone* | 1.12 | |  |  |  | 0.44 | .657 |
| **Zero-Inflated Model** |  | |  |  |  |  |  |
| (Intercept) | 1.28 | |  |  |  | 10.97 | **<.001** |

|  | **Random Effects** | | |
| --- | --- | --- | --- |
| σ^2^ | |  | 0.89 |
| τ_00_ _pp_ | |  | 0.29 |
| ICC | |  | 0.24 |
| N _pp_ | |  | 216 |
| Observations | |  | 432 |
| Marginal R^2^ / Conditional R^2^ | |  | 0.022/0.261 |
