# Supplementary material for: Putting the Social in Emotions: The Effect of Audience Presence on Pride and Embarrassment Across Ontogeny
Source: Dev Sci. 2025 May 19;28(4):e70024. doi: 10.1111/desc.70024 (PMC12087427; doi:10.1111/desc.70024)
Supplement: Supplementary file 2 — Supporting Information [file DESC-28-e70024-s001.docx]

| **Age Group** | **Audience Condition** | **Task** | ***N*** | ***M*_% SKT_** | ***SE*** |
| --- | --- | --- | --- | --- | --- |
| Adult | Alone | Emb | 36 | 0.13 | 0.03 |
| Adult | Alone | Pride | 36 | -0.01 | 0.03 |
| Adult | Audience | Emb | 37 | 0.22 | 0.04 |
| Adult | Audience | Pride | 37 | -0.01 | 0.04 |
| Older Child | Alone | Emb | 35 | 0.04 | 0.04 |
| Older Child | Alone | Pride | 35 | 0.04 | 0.04 |
| Older Child | Audience | Emb | 35 | 0.15 | 0.04 |
| Older Child | Audience | Pride | 35 | 0.12 | 0.04 |
| Younger Child | Alone | Emb | 31 | 0.12 | 0.05 |
| Younger Child | Alone | Pride | 31 | 0.18 | 0.06 |
| Younger Child | Audience | Emb | 29 | 0.10 | 0.03 |
| Younger Child | Audience | Pride | 29 | 0.14 | 0.04 |

**Table S1.**

Descriptive Statistics for Participants’ Cheek Temperature Change Scores Across Age Group, Audience Condition and Emotion Induction Task

*Note.* SKT = Skin Temperature (Cheek)

**Table S2.**

Descriptive Statistics for Participants’ Skin Conductance Level Change Scores Across Age Group, Audience Condition and Emotion Induction Task

*Note.* SCL = Skin Conductance Level.

| **Age Group** | **Audience Condition** | **Task** | ***N*** | ***M*_% Change SCL_** | ***SE*** |
| --- | --- | --- | --- | --- | --- |
| Adult | Alone | Emb | 36 | 12.69 | 2.85 |
| Adult | Alone | Pride | 36 | -4.68 | 1.24 |
| Adult | Audience | Emb | 37 | 9.26 | 2.17 |
| Adult | Audience | Pride | 37 | -2.59 | 1.51 |
| Older Child | Alone | Emb | 35 | 9.75 | 1.80 |
| Older Child | Alone | Pride | 35 | 0.13 | 1.72 |
| Older Child | Audience | Emb | 35 | 13.69 | 2.60 |
| Older Child | Audience | Pride | 35 | 2.40 | 1.67 |
| Younger Child | Alone | Emb | 33 | 10.48 | 2.83 |
| Younger Child | Alone | Pride | 33 | 7.14 | 2.34 |
| Younger Child | Audience | Emb | 34 | 7.03 | 2.51 |
| Younger Child | Audience | Pride | 34 | 5.04 | 2.26 |

**Table S3.**

Descriptive Statistics for Participants’ Heart Rate Variability Change Scores Across Age Group, Audience Condition and Emotion Induction Task

| **Age Group** | **Audience Condition** | **Task** | ***N*** | ***M*_% Change HRV_** | ***SE*** |
| --- | --- | --- | --- | --- | --- |
| Adult | Alone | Emb | 36 | -13.09 | 3.98 |
| Adult | Alone | Pride | 36 | -7.10 | 4.93 |
| Adult | Audience | Emb | 37 | -3.52 | 7.19 |
| Adult | Audience | Pride | 37 | -16.35 | 4.43 |
| Older Child | Alone | Emb | 35 | -3.17 | 3.81 |
| Older Child | Alone | Pride | 35 | -5.72 | 3.35 |
| Older Child | Audience | Emb | 35 | -5.40 | 6.39 |
| Older Child | Audience | Pride | 35 | -4.75 | 4.88 |
| Younger Child | Alone | Emb | 32 | 3.56 | 5.22 |
| Younger Child | Alone | Pride | 32 | 7.73 | 5.23 |
| Younger Child | Audience | Emb | 33 | 5.65 | 6.81 |
| Younger Child | Audience | Pride | 33 | 3.47 | 5.59 |

*Note.* HRV = Heart Rate Variability

**Table S4.**

Descriptive Statistics for Participants’ Embarrassment Nonverbal Expression Duration (Seconds) Across Age Group, Audience Condition and Emotion Induction Task

| **Age Group** | **Audience Condition** | **Task** | ***N*** | ***M*_Embarrassment_** | ***SE*** |
| --- | --- | --- | --- | --- | --- |
| Adult | Alone | Emb | 35 | 13.32 | 1.77 |
| Adult | Alone | Pride | 36 | 5.36 | 2.19 |
| Adult | Audience | Emb | 37 | 16.74 | 2.57 |
| Adult | Audience | Pride | 37 | 2.55 | 0.67 |
| Older Child | Alone | Emb | 35 | 22.23 | 2.84 |
| Older Child | Alone | Pride | 35 | 10.09 | 1.47 |
| Older Child | Audience | Emb | 35 | 20.12 | 2.46 |
| Older Child | Audience | Pride | 34 | 6.54 | 1.91 |
| Younger Child | Alone | Emb | 32 | 10.67 | 2.24 |
| Younger Child | Alone | Pride | 33 | 8.10 | 2.11 |
| Younger Child | Audience | Emb | 35 | 12.93 | 2.30 |
| Younger Child | Audience | Pride | 36 | 11.65 | 2.20 |

**Table S5.**

Descriptive Statistics for Participants’ Pride Nonverbal Expression Duration (Seconds) Across Age Group, Audience Condition and Emotion Induction Task

| **Age Group** | **Audience Condition** | **Task** | ***N*** | ***M*_Pride_** | ***SE*** |
| --- | --- | --- | --- | --- | --- |
| Adult | Alone | Emb | 36 | 0.55 | 0.22 |
| Adult | Alone | Pride | 36 | 1.06 | 0.75 |
| Adult | Audience | Emb | 37 | 0.65 | 0.38 |
| Adult | Audience | Pride | 37 | 0.72 | 0.65 |
| Older Child | Alone | Emb | 35 | 1.03 | 0.43 |
| Older Child | Alone | Pride | 35 | 0.89 | 0.36 |
| Older Child | Audience | Emb | 36 | 0.30 | 0.19 |
| Older Child | Audience | Pride | 36 | 0.78 | 0.38 |
| Younger Child | Alone | Emb | 35 | 0.49 | 0.20 |
| Younger Child | Alone | Pride | 35 | 0.65 | 0.23 |
| Younger Child | Audience | Emb | 37 | 0.61 | 0.27 |
| Younger Child | Audience | Pride | 37 | 0.25 | 0.12 |

**Table S6.**

Descriptive Statistics for Participants’ Self-Reported Embarrassment and Pride Across Age Group and Audience Conditions

| **Age Group** | **Audience Condition** | ***M_SubjPride_*** | ***SD_SubjPride_*** | ***M*_SubjEmb_** | ***SD*_SubjEmb_** |
| --- | --- | --- | --- | --- | --- |
| Adult | Alone | 1.11 | 0.58 | 0.86 | 0.68 |
| Adult | Audience | 0.97 | 0.50 | 1.19 | 0.57 |
| Older Child | Alone | 1.73 | 0.45 | 1.00 | 0.50 |
| Older Child | Audience | 1.69 | 0.47 | 1.11 | 0.53 |

*Note. Subj = Subjective Report*

**Figure S1**

*Bar Plots Indicating Cheek Temperature Change for Different Emotion Induction Tasks, Age Groups and Audience Conditions*


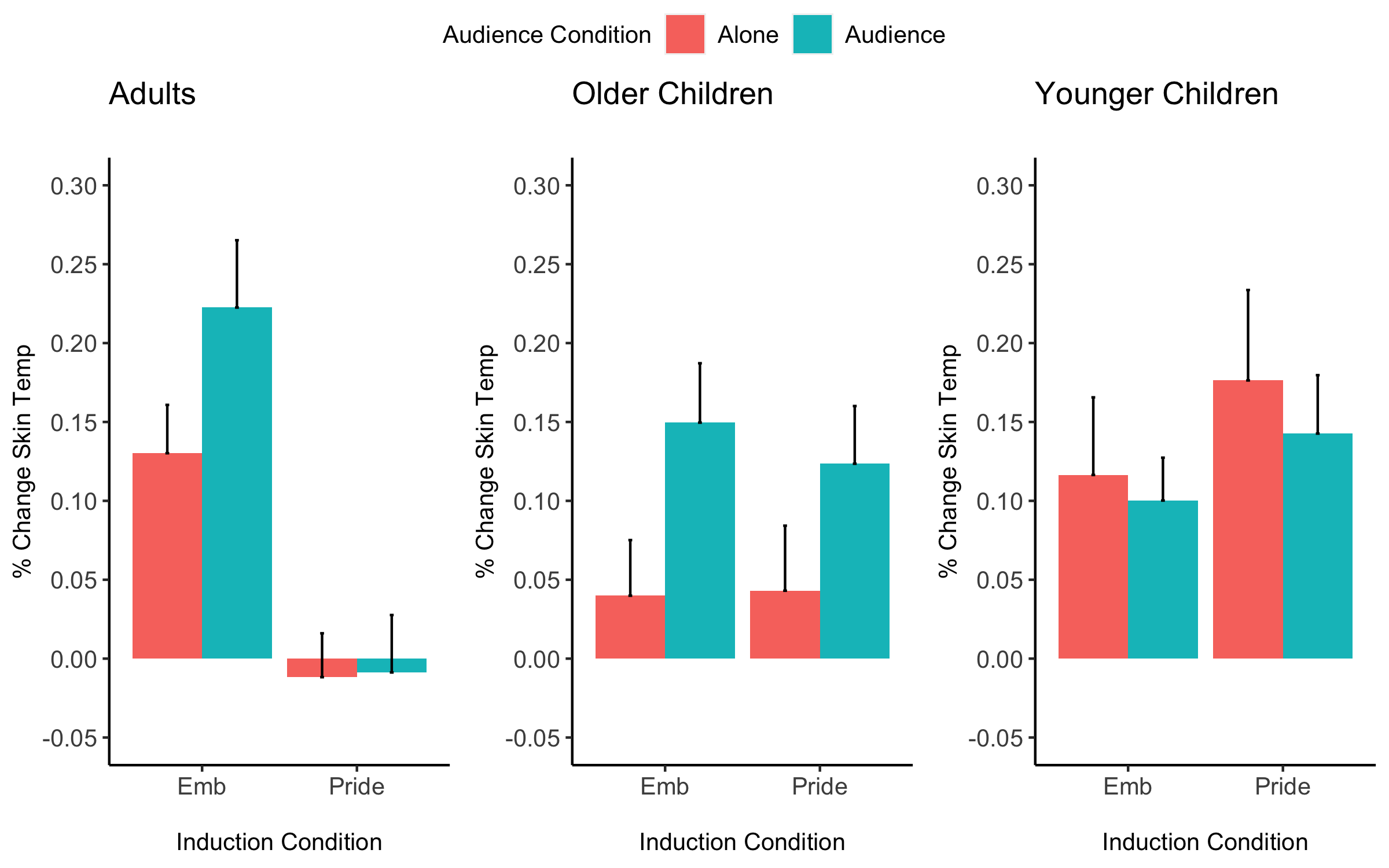


*Note.* Error bars represent standard error

**Figure S2**

*Bar Plots Indicating Skin Conductance Level Change for Different Emotion Induction Tasks, Age Groups and Audience Conditions*


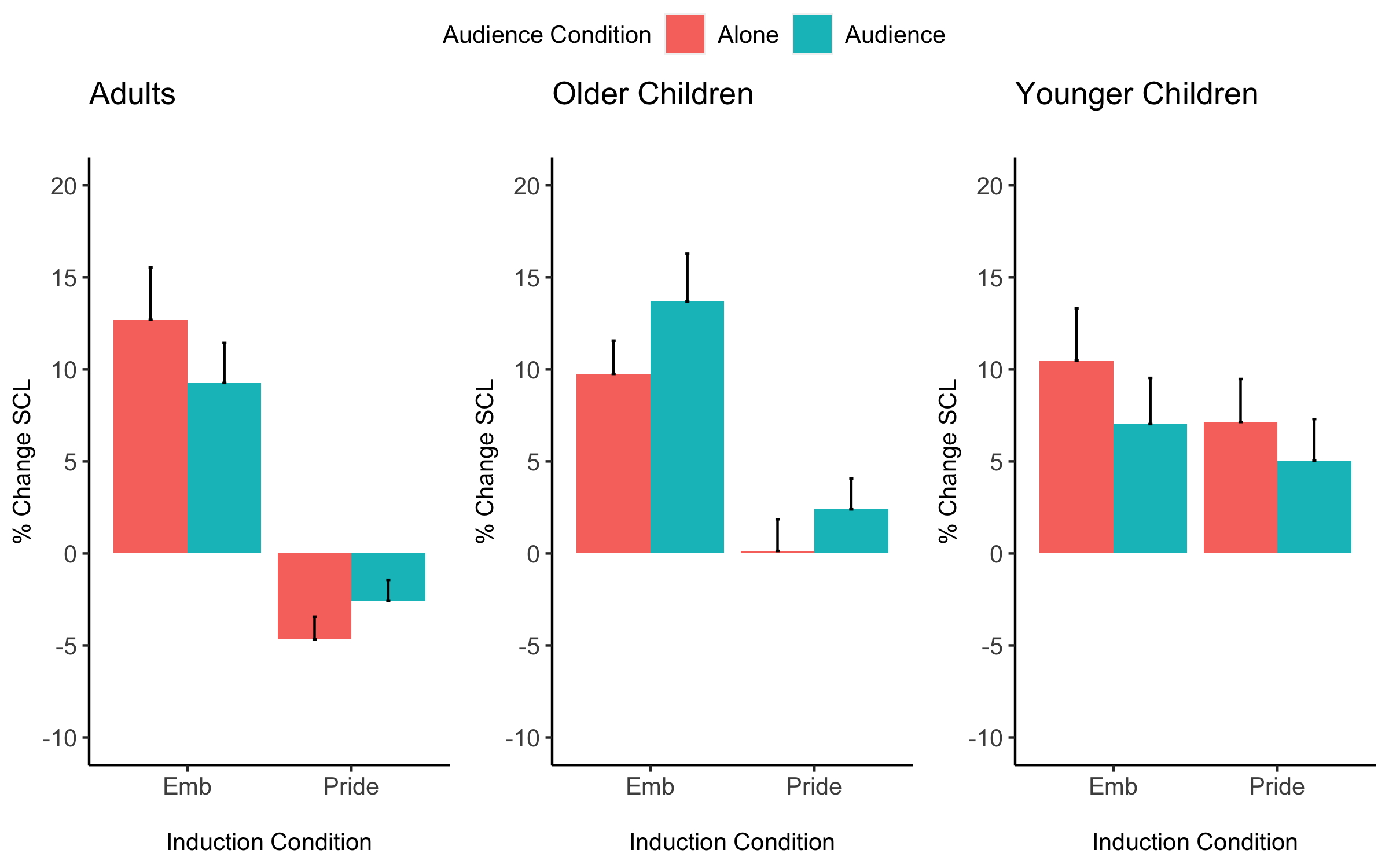


*Note.* Error bars represent standard error

**Figure S3**

*Bar Plots Indicating Heart Rate Variability Change for Different Emotion Induction Tasks, Age Groups and Audience Conditions*


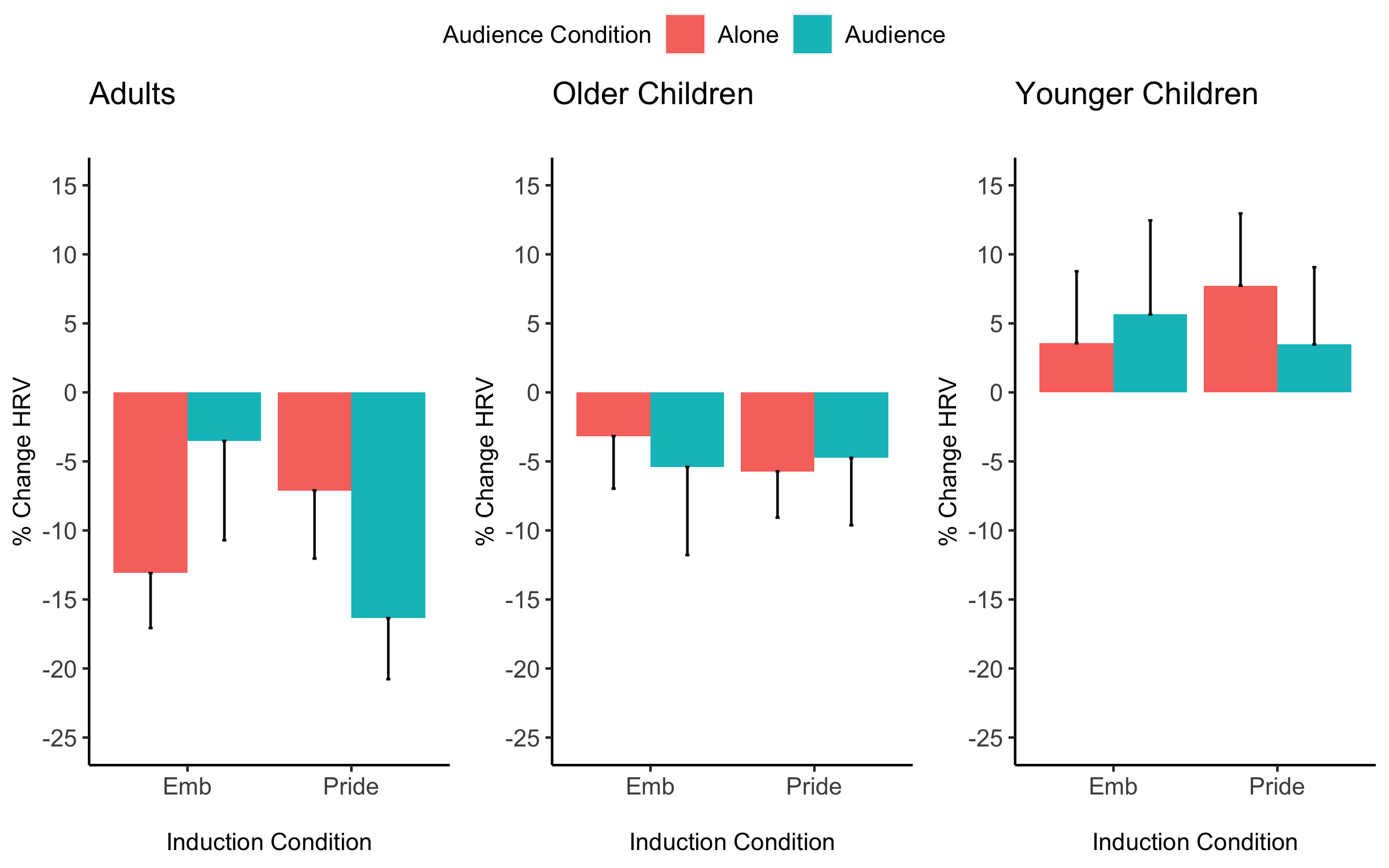


*Note.* Error bars represent standard error

**Figure S4**

*Bar Plots Indicating Total Displayed Nonverbal Expressions of Embarrassment for Different Emotion Induction Tasks, Age Groups and Audience Conditions*


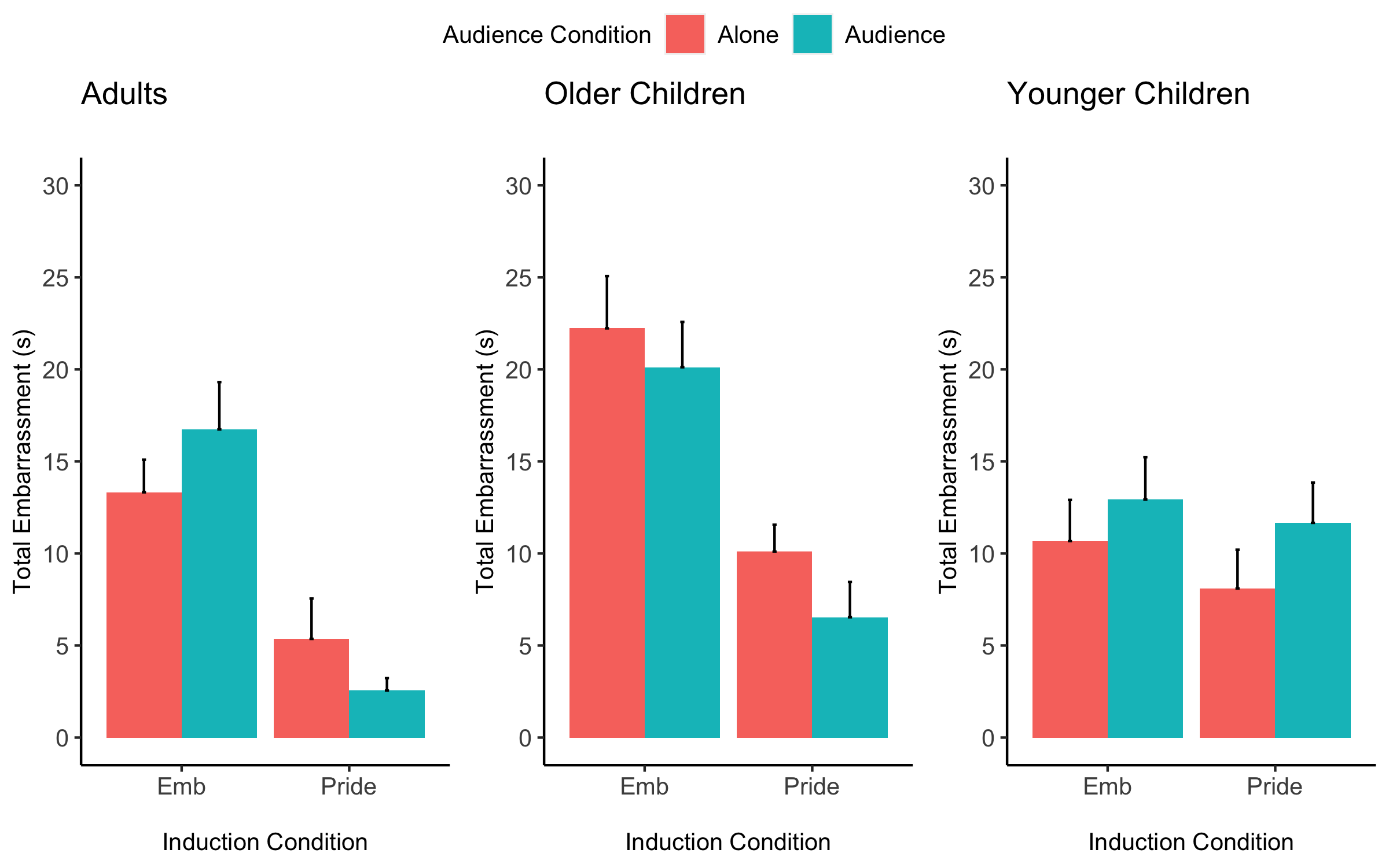


*Note.* Error bars indicate standard error
