# Supplementary material for: Putting the Social in Emotions: The Effect of Audience Presence on Pride and Embarrassment Across Ontogeny
Source: Dev Sci. 2025 May 19;28(4):e70024. doi: 10.1111/desc.70024 (PMC12087427; doi:10.1111/desc.70024)
Supplement: Supplementary file 3 — Supporting Information [file DESC-28-e70024-s003.docx]

**Table 1.** Correlation Table of all Dependent Variables in Each Task for Younger Children

**
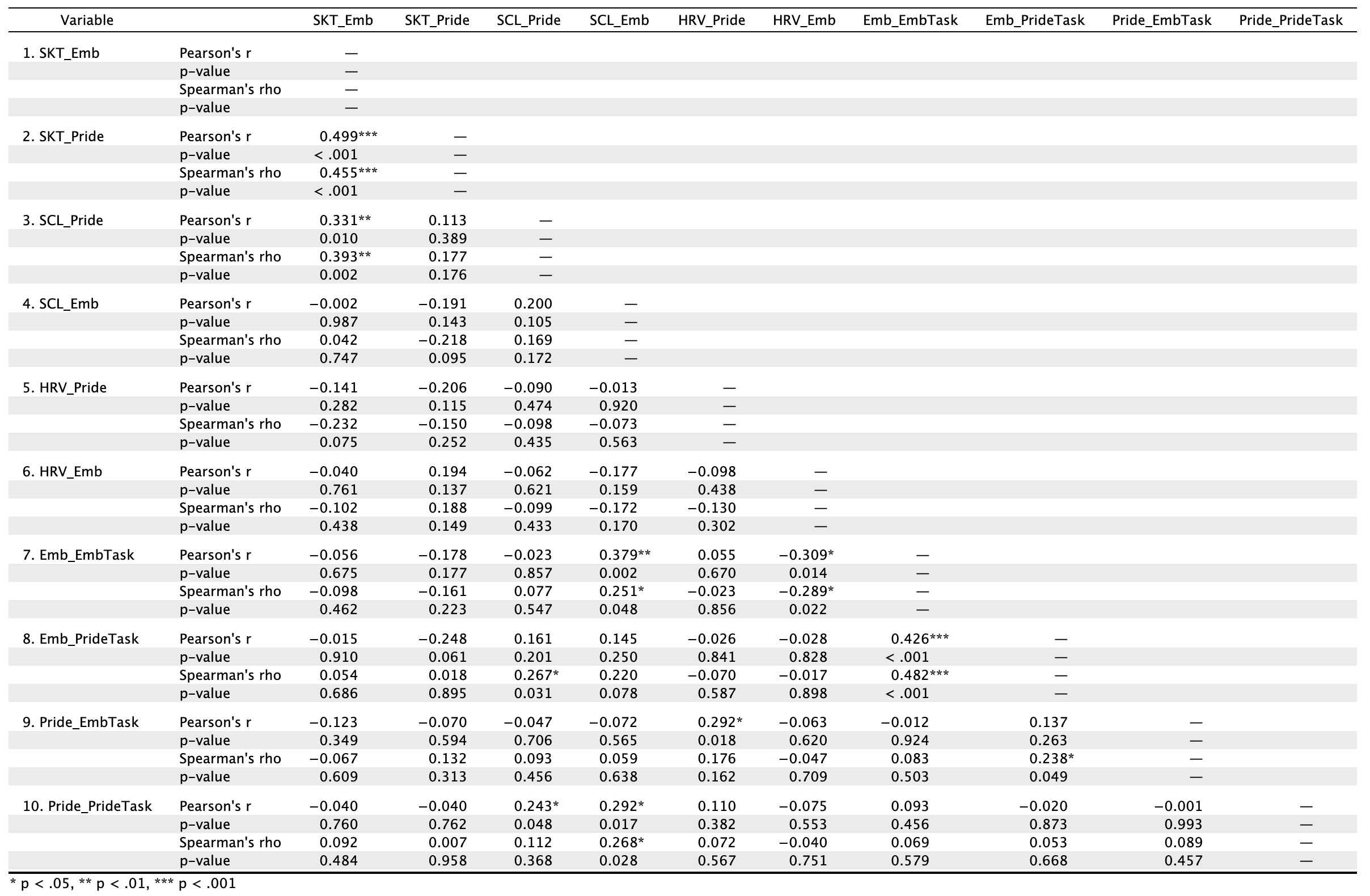
**

*Note.* SKT = Skin temperature, SCL = Skin conductance level, HRV = Heart rate variability, Emb_EmbTask refers to embarrassment nonverbal behavior in the viewing of the singing task, Emb_PrideTask refers to embarrassment in the viewing of the puzzle solving task

**Table 2.** Correlation Table of all Dependent Variables in Each Task for Older Children

**
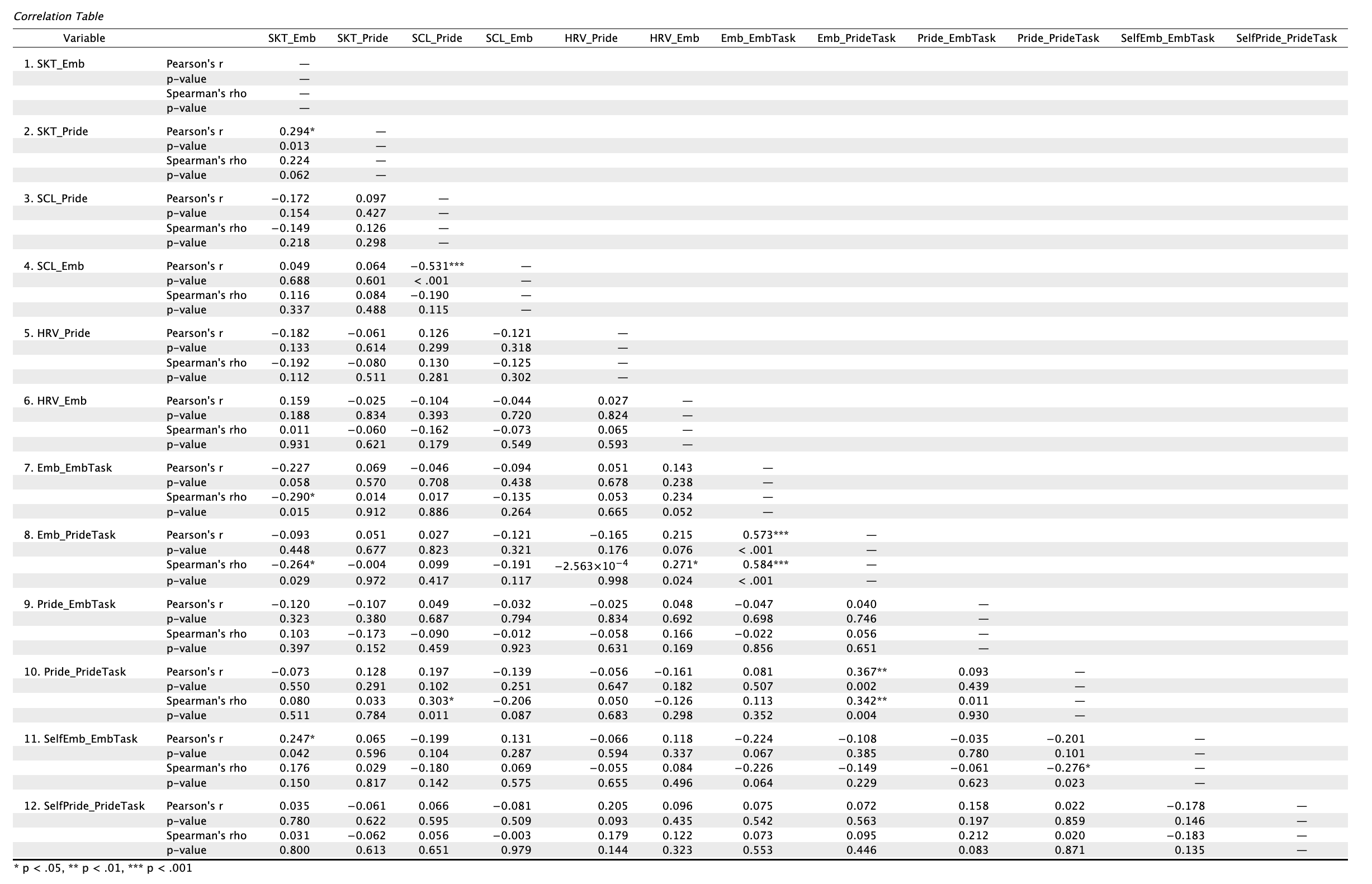
**

*Note.* SKT = Skin temperature, SCL = Skin conductance level, HRV = Heart rate variability

Emb_EmbTask refers to embarrassment nonverbal behavior in the viewing of the singing task, Emb_PrideTask refers to embarrassment in the viewing of the puzzle solving task (and vice versa)

SelfEmb SelfPride = Self-reported embarrassment/pride, respectively

**Table 2.** Correlation Table of all Dependent Variables in Each Task for Adults

**
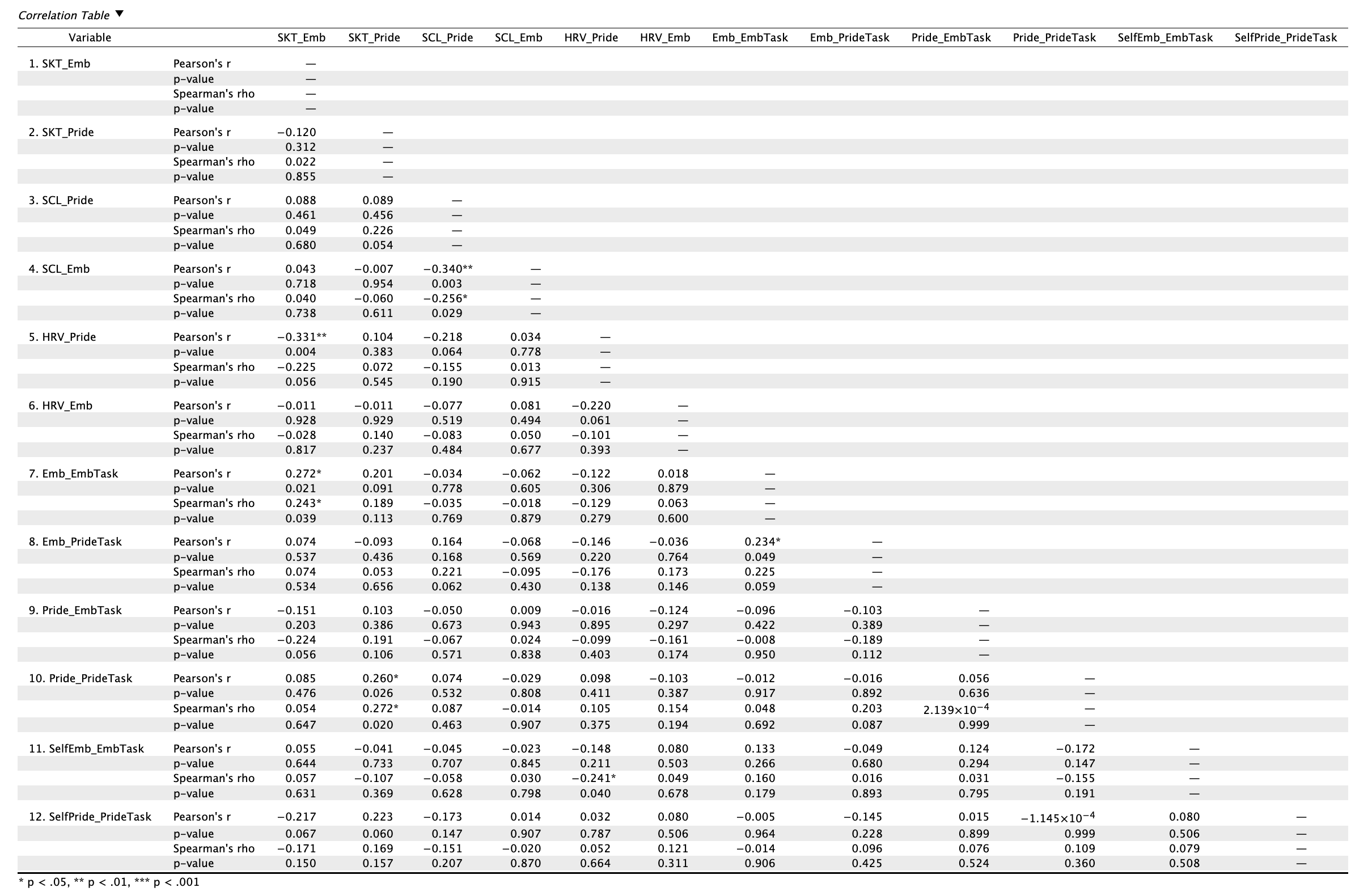
**

*Note.* SKT = Skin temperature, SCL = Skin conductance level, HRV = Heart rate variability

Emb_EmbTask refers to embarrassment nonverbal behavior in the viewing of the singing task, Emb_PrideTask refers to embarrassment in the viewing of the puzzle solving task (and vice versa)

SelfEmb SelfPride = Self-reported embarrassment/pride, respectively
