# Supplementary material for: Putting the Social in Emotions: The Effect of Audience Presence on Pride and Embarrassment Across Ontogeny
Source: Dev Sci. 2025 May 19;28(4):e70024. doi: 10.1111/desc.70024 (PMC12087427; doi:10.1111/desc.70024)
Supplement: Supplementary file 4 — Supporting Information [file DESC-28-e70024-s002.docx]

**Supplementary Materials 4 – Manipulation Checks**

***Baseline Physiological Arousal:***

1. Do we see increases in autonomic arousal when participants viewed the puzzle/singing task as compared to baseline? **Yes, we see the predicted pattern (increase SKT, increase SCL, decrease HRV).**

**Table 1.** Paired Samples T-Test [Task-Baseline] During the Viewing of the Puzzle Task

|  | | | | | | | | | | | | | | | |
| --- | --- | --- | --- | --- | --- | --- | --- | --- | --- | --- | --- | --- | --- | --- | --- |
| Measure 1 | |  | | Measure 2 | | t | | df | | p | | Cohen's d | | SE Cohen's d | |
| Cheek Task |  | - |  | Cheek Baseline |  | 3.999 |  | 202 |  | < .001 |  | 0.281 |  | 0.006 |  |
| SCL Task |  | - |  | SCL Baseline |  | 3.251 |  | 209 |  | 0.001 |  | 0.224 |  | 0.015 |  |
| HRV Task |  | - |  | HRV Baseline |  | -2.116 |  | 207 |  | 0.036 |  | -0.147 |  | 0.038 |  |
|  | | | | | | | | | | | | | | | |
| Note.  Student's t-test. | | | | | | | | | | | | | | | |

**Table 2.** Paired Samples T-Test [Task-Baseline] During the Viewing of the Singing Task

| \|  \| \| \| \| \| \| \| \| \| \| \| \| \| \| \| \| \| --- \| --- \| --- \| --- \| --- \| --- \| --- \| --- \| --- \| --- \| --- \| --- \| --- \| --- \| --- \| --- \| \| Measure 1 \| \|  \| \| Measure 2 \| \| t \| \| df \| \| p \| \| Cohen's d \| \| SE Cohen's d \| \| \| Cheek Task \|  \| - \|  \| Cheek Baseline \|  \| 7.419 \|  \| 202 \|  \| < .001 \|  \| 0.521 \|  \| 0.006 \|  \| \| SCL Task \|  \| - \|  \| SCL Baseline \|  \| 11.174 \|  \| 209 \|  \| < .001 \|  \| 0.771 \|  \| 0.017 \|  \| \| HRV Task \|  \| - \|  \| HRV Baseline \|  \| -3.501 \|  \| 207 \|  \| < .001 \|  \| -0.243 \|  \| 0.039 \|  \| \|  \| \| \| \| \| \| \| \| \| \| \| \| \| \| \| \| \| Note.  Student's t-test. \| \| \| \| \| \| \| \| \| \| \| \| \| \| \| \| |
| --- | --- | --- | --- | --- | --- | --- | --- | --- | --- | --- | --- | --- | --- | --- | --- | --- | --- | --- | --- | --- | --- | --- | --- | --- | --- | --- | --- | --- | --- | --- | --- | --- | --- | --- | --- | --- | --- | --- | --- | --- | --- | --- | --- | --- | --- | --- | --- | --- | --- | --- | --- | --- | --- | --- | --- | --- | --- | --- | --- | --- | --- | --- | --- | --- | --- | --- | --- | --- | --- | --- | --- | --- | --- | --- | --- | --- | --- | --- | --- | --- | --- | --- | --- | --- | --- | --- | --- | --- | --- | --- | --- | --- | --- | --- | --- | --- | --- | --- | --- | --- | --- | --- | --- | --- | --- | --- | --- | --- | --- | --- | --- | --- |

***Nonverbal Expressions of Embarrassment and Pride Within Tasks:***

1. Do participants show nonverbal expressions of pride and embarrassment above zero in the viewing of the *puzzle task*? **Yes – participants show above zero levels of both nonverbal embarrassment and pride.**

| **Table 3.** One Sample T-Test on the Mean Length of Nonverbal Embarrassment/Pride in the Viewing of the Puzzle Task | | | | | | | | | | | | |  |
| --- | --- | --- | --- | --- | --- | --- | --- | --- | --- | --- | --- | --- | --- |
|  | t | | df | | p | | Cohen's d | | | SE Cohen's d | | |  |
| Nonverbal Embarrassment |  | 9.563 |  | 209 |  | < .001 |  | 0.660 |  | | 0.076 |  | |
| Nonverbal Pride |  | 3.750 |  | 215 |  | < .001 |  | 0.255 |  | | 0.069 |  | |
|  | | | | | | | | | | | | |  |
| *Note.*  For the Student t-test, the alternative hypothesis specifies that the mean is different from 0. | | | | | | | | | | | | |  |
| *Note.*  Student's t-test. | | | | | | | | | | | | |  |

1. Do participants show nonverbal expressions of pride and embarrassment above zero in the viewing of the *singing task*? **Yes – participants show above zero levels of both nonverbal embarrassment and pride.**

| **Table 4.** One Sample T-Test on the Mean Length of Nonverbal Embarrassment/Pride in the Viewing of the Singing Task | | | | | | | | | | | | | | | | |  |
| --- | --- | --- | --- | --- | --- | --- | --- | --- | --- | --- | --- | --- | --- | --- | --- | --- | --- |
|  | | t | | | df | | | p | | | Cohen's d | | | SE Cohen's d | | |  |
| Nonverbal Embarrassment |  | | 15.974 |  | | 208 |  | | < .001 |  | | 1.105 |  | | 0.088 |  | |
| Nonverbal Pride |  | | 5.033 |  | | 215 |  | | < .001 |  | | 0.342 |  | | 0.070 |  | |
|  | | | | | | | | | | | | | | | | |  |
| *Note.*  For the Student t-test, the alternative hypothesis specifies that the mean is different from 0. | | | | | | | | | | | | | | | | |  |
| *Note.*  Student's t-test. | | | | | | | | | | | | | | | | |  |

***Nonverbal Expressions of Embarrassment and Pride Between Tasks:***

1. Do participants show more nonverbal expression of pride in the viewing of the puzzle task compared to the viewing of the singing task? **No, no differences in nonverbal pride expressions (0= no pride, 1= pride) across conditions.**

**Table 5.** Contingency Table Displaying Distribution of Nonverbal Pride Across Tasks

|  | | | | | | | |
| --- | --- | --- | --- | --- | --- | --- | --- |
|  | | Viewing Task | | | |  | |
| Nonverbal Pride | | Singing | | Puzzle | | Total | |
| 0 |  | 168 |  | 170 |  | 338 |  |
| 1 |  | 48 |  | 46 |  | 94 |  |
| Total |  | 216 |  | 216 |  | 432 |  |
|  | | | | | | | |

| **Table 6.** Chi-Squared Tests of Equivalence of Nonverbal Pride Across Tasks | | | | | | | |
| --- | --- | --- | --- | --- | --- | --- | --- |
|  | | Value | | df | | p | |
| Χ² |  | 0.054 |  | 1 |  | 0.816 |  |
| N |  | 432 |  |  |  |  |  |
|  | | | | | | | |

1. Do participants show more nonverbal expression of embarrassment in the viewing of the puzzle task compared to the viewing of the singing task? **Yes, participant show more nonverbal expressions of embarrassment (0 = no embarrassment, 1 = embarrassment) in the viewing of the singing task compared to the viewing of the puzzle task.**

| **Table 7.** Contingency Table Displaying Distribution of Nonverbal Embarrassment Across Tasks | | | | | | |  |
| --- | --- | --- | --- | --- | --- | --- | --- |
|  | | **Viewing Task** | | | |  |  |
| Nonverbal Embarrassment | | Singing | | Puzzle | | Total |  |
| 0 |  | 15 |  | 46 |  | 61 |  |
| 1 |  | 193 |  | 164 |  | 357 |  |
| Total |  | 208 |  | 210 |  | 418 |  |
|  | | | | | | |  |
|  | | | | | | |  |

| **Table 8.** Chi-Squared Tests of Equivalence of Nonverbal Embarrassment Across Tasks | | | | | | |  |
| --- | --- | --- | --- | --- | --- | --- | --- |
|  | | Value | | df | | p |  |
| Χ² |  | 18.101 |  | 1 |  | < .001 |  |
| N |  | 418 |  |  |  |  |  |
|  | | | | | | |  |
